# Platelet-derived microparticles provoke chronic lymphocytic leukemia malignancy through metabolic reprogramming

**DOI:** 10.3389/fimmu.2023.1207631

**Published:** 2023-06-27

**Authors:** Ehsan Gharib, Vanessa Veilleux, Luc H. Boudreau, Nicolas Pichaud, Gilles A. Robichaud

**Affiliations:** ^1^ Department of Chemistry and Biochemistry, Université de Moncton, Moncton, NB, Canada; ^2^ Atlantic Cancer Research Institute, Moncton, NB, Canada; ^3^ New Brunswick Center for Precision Medicine, Moncton, NB, Canada

**Keywords:** platelets, microvesicles, leukemia, microparticles, extracellular vesicles, mitochondria

## Abstract

**Background:**

It is well established that inflammation and platelets promote multiple processes of cancer malignancy. Recently, platelets have received attention for their role in carcinogenesis through the production of microvesicles or platelet-derived microparticles (PMPs), which transfer their biological content to cancer cells. We have previously characterized a new subpopulation of these microparticles (termed mito-microparticles), which package functional mitochondria. The potential of mitochondria transfer to cancer cells is particularly impactful as many aspects of mitochondrial biology (i.e., cell growth, apoptosis inhibition, and drug resistance) coincide with cancer hallmarks and disease progression. These metabolic aspects are particularly notable in chronic lymphocytic leukemia (CLL), which is characterized by a relentless accumulation of proliferating, immunologically dysfunctional, mature B-lymphocytes that fail to undergo apoptosis. The present study aimed to investigate the role of PMPs on CLL metabolic plasticity leading to cancer cell phenotypic changes.

**Methods:**

CLL cell lines were co-incubated with different concentrations of human PMPs, and their impact on cell proliferation, mitochondrial DNA copy number, OCR level, ATP production, and ROS content was evaluated. Essential genes involved in metabolic-reprogramming were identified using the bioinformatics tools, examined between patients with early and advanced CLL stages, and then validated in PMP-recipient CLLs. Finally, the impact of the induced metabolic reprogramming on CLLs’ growth, survival, mobility, and invasiveness was tested against anti-cancer drugs Cytarabine, Venetoclax, and Plumbagin.

**Results:**

The data demonstrated the potency of PMPs in inducing tumoral growth and invasiveness in CLLs through mitochondrial internalization and OXPHOS stimulation which was in line with metabolic shift reported in CLL patients from early to advanced stages. This metabolic rewiring also improved CLL cells' resistance to Cytarabine, Venetoclax, and Plumbagin chemo drugs.

**Conclusion:**

Altogether, these findings depict a new platelet-mediated pathway of cancer pathogenesis. We also highlight the impact of PMPs in CLL metabolic reprogramming and disease progression.

## Introduction

Chronic lymphocytic leukemia (CLL) is the most prevalent adult blood cancer in western societies and consists of almost 7% of non-Hodgkin lymphomas (NHL) cases worldwide. CLL is defined by uncontrollable proliferation of monoclonal lymphocytes, eventually resulting in a growing number of low or nonfunctional B-cells. Generally, CLL affects older adults (median age = 70) where approximately 80% of diagnosed cases sustain a five-year survival incidence. Although CLL and its prognosis are classified by several genetic factors (i.e., ZAP-70 mutation (1), immunoglobulin variable region heavy chain (IGHv) status (2), and β2 microglobulin levels (3)), CLL is not associated with a specific cytogenetic or molecular defect. In addition, while available treatments generally induce remission, most patients recidivate due to chemoresistance (4). Consequently, CLL still remains an incurable disease today.

In addition to genetic lesions, several pathogenic mechanisms have been shown to be key regulators of CLL disease, such as cancer cell interactions with its microenvironment to induce cell adaptability during disease progression. This environmental adaptation is conveyed through cancer cell metabolic reprogramming, which cumulates to promote cell survival, proliferation, and immune escape (4–6). This metabolic plasticity is particularly vital for CLL cells residing in different compartments such as bone marrow, spleen, lymph nodes, and peripheral blood, which reflect its pathological features (i.e., marrow infiltration, splenomegaly lymphadenopathy and lymphocytosis respectively) with the disease (7). Previous studies have reported that aerobic glycolysis is the dominant metabolic pathway during the early stages of CLL disease in which it efficiently supports cell growth by rapid ATP production and enhanced tissue disruption (8). However, the metabolic status of CLL is altered upon entering the bloodstream by an increase of mitochondrial content and subsequent activation of oxidative phosphorylation (OXPHOS) to evade immune assault (5, 9, 10). These changes also result in abundant reactive oxygen species (ROS) production, leading to chronic oxidative stress and metastasis stimulation (8, 11). Although metabolic plasticity is a well characterized hallmark of cancer progression, the exact mechanisms and cues involved in metabolic remodeling during cancer pathogenesis have not been fully elucidated to this day. Nonetheless, mitochondrial mass and function have been intimately linked to CLL disease progression (11–15).

Inflammation is another critical component of cancer malignancy (16) where platelets are particularly active in these events (17–19). Platelets are generated from megakaryocytes found in the bone marrow and are released in the bloodstream where they represent the most abundant blood cell type after erythrocytes (20). More recently, platelets have garnered attention for their role in cancer through the production of platelet-derived microparticles (PMPs). Upon activation, platelets shed and release extracellular vesicles in the extracellular milieu known as microparticles (also termed microvesicles, 0.1 –1 μm) (21). Moreover, PMPs and their packaged bioactive content (i.e., RNAs, transcription factors, inflammatory enzymes, etc.) are horizontally transferred into recipient target cells upon vesicle internalization (22, 23). It has been shown that the PMPs can cargo more than 600 proteins involved in cellular process (26%), regulation (22%), interaction with cells and organisms (9%), metabolic process (8%), response to stimulus and developmental process (6%), localization (5%), immunosystem process (4%), and reproduction (2%) (24). Meanwhile, the cytoplasm section had the most significant number of proteins (20%), followed by the plasma membrane (14%), other intracellular organelles (11%), nucleus (10%), cytoskeleton (9%), mitochondria (6%), extracellular and macromolecular complex (5%), endoplasmic reticulum (4%), endosome (3%), and cell surface (1%) (24). More than half of the identified proteins like STXBP2 and SH3BGRL had binding activity (48%), catalytic activity (29%) like PP2B and Catalase, enzyme regulator activity (5%), structural molecular activity (5%), molecular transducer activity, and antioxidant activity (2%).

Microparticles represent physiological vehicles of bioactive material for intercellular communication (25, 26). Given that PMPs constitute 70-90% of all circulating microparticles (27, 28), they have also been associated with numerous diseases including multiple sclerosis (29, 30), arthritis (31, 32), atherosclerosis (33, 34), and cancer (35, 36). The exact mechanism by which PMPs stimulate gene transcription for metabolic genes has not been fully understood yet, there are several proposed mechanisms such as (37–42): *i*) epigenetic modifications in which PMPs can transfer microRNAs (miRNAs) to recipient cells to regulate gene expression and modify the epigenetic landscape of the recipient cells through DNA methylation and histone modifications, thereby stimulating or inhibiting gene transcription; *ii*) signaling pathways activated by PMPs including PI3K-Akt and MAPK, which activate transcription factors (ex: nuclear factor kappa B (NF-κB)) that bind gene promoters to activate transcription; *iii*) transcription factor transfer through PMP cargo, which regulate recipient cell gene transcription (37–42). Yet, further research is needed to fully understand the mechanisms by which PMPs modulate gene transcription and how these processes contribute to cellular metabolism.

We have previously identified and reported a novel subpopulation of PMPs which retains functional mitochondria (25, 32). Although the internalization of mitoMPs leads to a net increase of total intracellular mitochondrial mass in recipient cells, the net phenotypic outcome in permissive cells remains to be investigated^33^. This phenomenon is of particular interest in cancer as many facets of mitochondrial physiology, beyond ATP production, support cancer processes such as proliferation, redox homeostasis, invasion, drug resistance, and apoptosis resistance (43–47). Given the predominant role of mitochondria in cancer processes, we therefore set out to investigate whether and how a gain in additional mitochondrial mass (and mitochondrial function) supplied by PMPs, would provide CLL cells with a significant biological benefit leading to disease progression. In this study, we demonstrate a new aspect in the complex communication network between CLL cells and blood platelets as a source of foreign mitochondrial supply for cancer cells. We found that CLL cell acquisition of PMP-sourced mitochondria leads to a positive impact in cancer metabolism. Specifically, co-cultures of CLL with PMPs elicited metabolic reprogramming resulting in increased levels of oxygen consumption rates (OCR), ATP levels, and production of ROS. CLL cells treated with PMPs are also characterized by enhanced proliferation, mobility, invasiveness, and anticancer drug resistance. Our study thus characterizes new leukemic cell interactions with its microenvironment enabling cancer cell metabolic reprogramming and enhanced malignant features. These findings also identify new platelets mechanisms supporting cancer malignancy, which concomitantly provide new avenues for the development of anticancer strategies.

## Materials and methods

### Cell culture and treatments

Human leukemic cell lines CII (#ACC 773), MEC-1 (ACC 497), and the megakaryocytic Set2 cell line (#ACC 608) were purchased from the Leibniz Institute DSMZ-German Collection of Microorganisms and Cell Culture (DSMZ Braunschweig, Germany). The cells were grown and maintained in RPMI 1640 media (ThermoFisher Scientific, Burlington, ON, Canada) supplemented with 10% fetal bovine serum/FBS (Wisent Bioproducts, St‐Bruno, QC, Canada), FBS) at 37°C under 5% CO_2_ in a humified incubator. Set2 cells were grown in RPMI 1640 medium supplemented with 20% fetal bovine serum. Culturing of cells was performed at a medium density of 2-5 x 10^5^ cells/mL for CII and 0.5-2.0 x 10^6^ cells/mL for MEC-1 and split at an appropriate ratio of 1:2 every 40 hrs. Cell treatments for phenotypic analyses included anticancer drug compounds Cytarabine, Venetoclax, and Plumbagin (Sigma-Aldrich) at the indicated concentrations for the indicated time points. To evaluate PMP internalization levels into cells, co-cultures were performed with cells labeled with anti-human CD19-FITC antibody (BioLegend, San Diego, CA, USA) and MitoTracker^®^ Deep Red (MTDR, ThermoFisher Scientific)-labeled PMPs followed by cell cytometry using the 644 nm EX for MTDR and 488 nm EX for CD19 detection.

### Isolation of platelets and platelet-derived microparticles

This study was conducted in accordance with the guidelines of the Declaration of Helsinki of 1975 (revised 2013, https://www.wma.net/what-we-do/medical-ethics/declaration-of-helsinki/). Institutional Review Board Statement and approval for studies involving human samples have been granted from both the Vitalité Health Network (project: CER7-3-17 Ver. 5) and the Université de Moncton (NB, Canada) (project: 1516-002) research ethics boards. Peripheral blood was obtained from consenting healthy volunteers (three men and seven women, aged 22-37). Healthy donors selected did not have any known autoimmune diseases and did not previously receive any preoperative chemotherapy and/or radiotherapy.

Patient samples consisted of 200 mL of whole peripheral blood, collected from cubital vein using a 21-gauge needle and Vacutainer tubes (ThermoFisher Scientific) containing the anticoagulant acid citrate dextrose (ACD; 0.085 mM sodium citrate, 0.0702 mM citric acid, 0.111 mM dextrose, pH 4.5). The platelets-rich plasma (PRP) layer was separated from the erythrocytes and other cell types by centrifugation at room temperature for 10 min at 265 x *g.* The PRP was transferred to a new tube where ACD (1:5 of the initial PRP volume) and ethylenediaminetetraacetic acid (EDTA, 10 mM, pH 8.0) were added, and the mixture was subsequently centrifuged at 400 x *g* for 2 minutes at room temperature. The supernatant was collected into a new tube and centrifuged at 1,300 x *g* for 10 minutes at room temperature. The harvested pellets were gently suspended in 200 μL Tyrode buffer (134 mM NaCl, 2.9 mM KCl, 0.34 mM Na_2_HPO_4_, 12 mM NaHCO_3_, 20 mM HEPES, 1 mM MgCl_2_, 5 mM glucose, and 0.5 mg/mL bovine serum albumin (BSA), pH 6.5) and then transferred in 25% volume of initial PRP of Tyrode solution (pH 7.4). Platelet count was performed with a hemocytometer slide under a Nikon TMS inverted phase-contrast microscope equipped with a Coolpix 5000 high-resolution digital camera (Nikon Corp, Melville, NY, USA). PMP production was initiated by incubating platelets (3 x 10^8^ cells/mL) with thrombin (0.1 U/mL, Sigma-Aldrich, Oakville, ON, Canada) and 5 mM CaCl_2_ overnight at room temperature. The next day, EDTA (10 mM) was added to stop the reaction and remaining platelets were isolated by centrifugation at 500 x *g* for 10 min. The supernatant was collected and centrifuged at 17,800 x *g* for 90 min at room temperature. Pelleted PMPs were then resuspended in phosphate-buffered solution (PBS, Sigma-Aldrich).

PMP quantification were performed by platelet fluorescence labeling with either 100 nM MTDR (mitochondria staining) with 2 μM PKH67 (Sigma-Aldrich) (cell membrane staining), or anti-CD41-FITC (BioLegend) (membrane staining), followed by an incubation for 15 min at 37°C. The labeling process was stopped by adding 10 mM EDTA and subsequent centrifugation at 1,300 x *g* for 10 min. The pellet, containing labelled PMPs, was resuspended in Tyrode buffer and characterized by flow cytometry (Attune NxT Flow Cytometer, ThermoFisher Scientific) using the 644 nm excitation (EX) and 665 nm emission (EM) for MTDR and 490/502 nm for PKH67. All solutions were filtered with a 0.2 µm filter before use. All experiments were performed with freshly isolated PMPs and were never frozen to ensure the functional integrity of mitochondria, unless otherwise indicated.

### Mitochondrial isolation and transfections

Mitochondrial isolation was performed using a PIERCE Mitochondria Isolation Kit (ThermoFisher Scientific) according to the manufacturer’s instructions. A total of 2 × 10^7^ cells were pelleted by centrifuging at 850 × *g* for 2 min and subsequently treated with 800 μL of Mitochondria Isolation Reagent A, vortexed and kept for 2 min on ice. The samples were then mixed with 10 μL of Mitochondria Isolation Reagent B and chilled for 5 min. At the end of incubation, cells received 800 μL of Mitochondria Isolation Reagent C and centrifuged at 700 × *g* for 10 min at 4°C. The supernatant was then collected and centrifuged at 12,000 × *g* for 15 min at 4°C. The obtained pellet was mixed with 500 μL of Mitochondria Isolation Reagent C and centrifuged again at 12,000 × *g* for 5 min. The purified mitochondria were kept on ice before downstream handling.

Upon isolation, mitochondria were labeled with MTDR and quantified using flow cytometry. Purified mitochondria were then mixed with PBS and co-cultured with cells labeled with DAPI and MitoTracker™ Green FM (MTG, ThermoFisher Scientific) at 10 mitochondria per cell (10:1), and 100 mitochondria per cell (100:1) ratios as previously instructed (48, 49). Following a 24- and 48-hour incubation, cell media was replaced with fresh RPMI-1640 supplemented with 10% FBS. Cells were monitored using an Olympus FV1000 confocal fluorescence microscope at 600x magnification (UPLAN 60x oil, 1.35NA, Olympus) with appropriate excitation laser and filters. Confocal microscopy fluorescence was then confirmed by flow cytometry where gating events were based on CD19 expression and MTDR-labeled mitochondria internalization.

### Cell proliferation assays

Cell viability was measured by the Cell Titer Blue assay (Promega, Madison, WI, USA) based on the manufacturer’s guidelines. Briefly, cells were seeded in a 96-wells plate at a density of 5 x 10^3^ cells/well and treated with PMPs (10 PMP/cell) for the indicated time points. Thereafter, 20 μL of Cell Titer Blue substrate was added to each well and incubation under standard cell culture conditions for 1 hour. The plate was read with a Synergy H1 Hybrid Multi-Mode Microplate Reader (Agilent, Santa Clara, CA, USA) at Ex/Em: 560/590 nm *via* Gen5 data analysis software (Version 2.01).

### Cell-cycle analysis

Cell-cycle progression was evaluated with a propidium iodide (PI) solution (MilliporeSigma, Burlington, Massachusetts, USA). Briefly, 5 x 10^6^ cells were collected by centrifugation at 300 x *g* for 5 min, washed in 1 mL PBS buffer containing 2% FBS, and resuspended in 5 mL chilled 70% ethanol dropwise and kept at 4°C. After a 24-hour incubation, fixed cells were centrifuged at 850 x *g* for 10 min, PBS-washed, and resuspended in a staining mixture of 1 mL PI staining solution + 50 µL RNase A (New England Biolabs, Whitby, Ontario, Canada) at 4°C overnight. Samples were then analyzed by flow cytometry at Ex/Em: 536/617 nm using the FlowJo software (Version 10.7, Tree Star, Inc., Ashland, OR., USA).

### Apoptosis assays

Apoptosis was measured using the FITC Annexin-V Apoptosis Detection Kit with PI (BioLegend). In accordance with the manufacturer’s protocol, 100 μL of PBS-washed cell samples were diluted in Annexin-V Binding Buffer at a final concentration of 1 × 10^7^ cells/mL and subsequently treated with 5 μL of FITC Annexin-V + 10 µL of PI solution for 15 min at room temperature in the dark. After incubation, 400 µL of Annexin-V Binding Buffer was added to each sample and then analyzed by flow cytometry.

### Oxygen consumption rate analysis

Cellular oxygen consumption was measured using high-resolution respirometry (Oxygraph-2k, Oroboros Instruments, Innsbruck, Austria) as previously described (50). Briefly, oxygen calibration was performed at 37°C with a continuous stirring speed of 750 rpm prior to measurements of mitochondrial OCR using 2.5 x 10^6^ cells mixed with 2 mL of complete media. Evaluation of physiological ROUTINE respiration was determined as the basal state of cellular oxygen consumption (i.e., with endogenous substrates). The non-phosphorylating resting state (LEAK respiration) was obtained after injection of oligomycin (Omy; 2.5 µM) to inhibit ATP synthase. The uncoupled respiratory capacity (electron transport system capacity*;* ETS) was determined as the maximal capacity of the mitochondrial ETS and was measured by adding sequential injections of carbonyl cyanide m-chlorophenyl hydrazone (CCCP; 0.5 µM steps) until no further increase in OCR was detected. Residual oxygen consumption (ROX) was subsequently obtained with injections of rotenone (ROT; 0.5 µM) to inhibit complex I and antimycin A (AmA; 2.5 µM) to inhibit complex III (50, 51). All inhibitors were purchased from Sigma-Aldrich. OCRs were corrected with ROX values and analyzed using DatLab software 7.2 (Oroboros Instruments).

### ATP analysis

Cellular ATP levels were determined by an ATP determination kit (ThermoFisher Scientific) according to manufacturer’s instructions. Briefly, 1 x 10^4^ cells were seeded in 96-well plates with fresh media supplemented with 10% FBS for 1 hour. For glycolytic ATP, cells were treated for 15 min at 37°C with ROT (0.5 μM) and AmA (2.5 μM) to inhibit mitochondrial complexes I and III, respectively. Content of the wells was then collected by centrifugation at 500 x *g* for 5 min at 4°C, PBS-washed, and centrifuged again. Cells were lysed in 1% Triton for 10 min at room temperature and centrifuged for 5 min at 500 x *g*. Then, 10 μL of each cell lysate were transferred to another 96-well plate and diluted with 90 μL of the kit’s Luciferine-Luciferase reaction reagent loaded withing the injector of a Synergy H1 Hybrid Multi-Mode Microplate Reader (Agilent). Luminescence was obtained with 1 sec integration times and analyzed with the Gen5 data analysis software (Agilent).

### ROS assessment

The CellROX^®^ Green Flow Cytometry Assay Kit (ThermoFisher Scientific) and the MitoSOX™ Red mitochondrial superoxide indicator (ThermoFisher Scientific) were used for intracellular ROS and mitochondrial superoxide determination, respectively. To this point, 5 x 10^5^ cells/well were added to 24-well plates and treated with PMPs as described previously. N-acetylcysteine (NAC) and Tert-butyl hydroperoxide (TBHP) were used as negative and positive controls, respectively. Following co-culturing conditions, cells were PBS-washed three times and kept in 1 mL of media containing one of the following ROS inducers: *i*) 6 μM AmA + 5 μM ROT; *ii*) phorbol 12-myristate 13-acetate (PMA, 80 ng) for 15 min; or *iii*) mixture of AmA + ROT + PMA for 30 min. Then, each well received 1 μM/mL CellROX green reagent for 45 min along with 5 μM SYTOX Red Dead Cell Stain (1μl/mL) for 15 min. The samples were then analyzed by flow cytometry at Ex/Em: 508/525 nm (CellROX green) and 640/658 nm (SYTOX Red Dead Cell Stain). As for the evaluation of mitochondrial superoxide, 1 x 10^5^ cells/mL from each experimental group were mixed with 5 μM MitoSOX™ reagent working solution and kept from the light for 10 min at 37°C. The samples were then analyzed by flow cytometry at Ex/Em: 510/580 nm.

### Real-time PCR

Total RNA was extracted from the cell samples using TRIzol^®^ reagent (ThermoFisher Scientific) and treated with DNase-I for 1 hour (Sigma-Aldrich). The integrity of RNA samples was estimated using a Nanodrop ND-1000 spectrophotometer (Nanodrop Technologies Wilmington, DE, USA). First-strand cDNA was synthesized with the SuperScript III Reverse Transcriptase (ThermoFisher Scientific) based on the manufacturer’s recommendations.

Real-time PCR analysis of target genes was carried out on a CFX Connect™ Real-Time System (Bio-Rad Laboratories, Hercules, CA, USA) with the PerfeCta SYBR Green Supermix kit (Quanta Biosciences, Beverly, CA, USA). Reaction mixtures were incubated at 95°C for 5 min, followed by 39 cycles consisting of 94°C for 15 sec, 52°C for 30 sec, and 72°C for 30 sec. Amplifications were analyzed with the Bio-Rad CFX Maestro software (Bio-Rad Laboratories) and normalized to human 18S rRNA using the 2^-ΔΔCT^ formula (52). Primers are shown in **Table 1**.

**Table 1:** Primer sequences.

Gene	Forward (5’→3’)	Reverse (5’→3’)
**HIF1-A**	CTCTGATCATCTGACCAAAACTCA	CAACCCAGACATATCCACCTC
**ACSL1**	GTGAAAGCAACAGAGAATACAGTC	TCCATGACACAGCATTACACA
**ACSL3**	ATCTGTTTCTGCTGTCCTGTT	AACTAATGGTGCTCCCACTC
**ACSL4**	TCATGTGCTAGAACTGACAGC	GTACAGTCTCCTTTGCTTCCTT
**ACACA**	CTGCCCACATCTCATCCAAA	GTACATCGCTGACACTAGCTAC
**ACLY**	AGGACAGGAGCTCATCTACG	GTCACCATCAGACACATCTCA
**ASPH**	CATCTGTAGCTGTCGTTTGGT	CCACATCAAAATCTCCATCACC
**FASN**	TTTGATGCCTCCTTCTTCGG	CGGAGTGAATCTGGGTTGATG
**FH**	AGGTCTGGGAGAATTGATCTTG	TCCGACAGTGACAGCAAC
**FTO**	TCTTGACTGCCATCCTTGC	CCGACATTCTGGCTTCTGAT
**IDH**	ACTATGATGGTGACGTGCAG	CTGCTTCTACTGTCTTGCCA
**MCL1**	CATTAGCAGAAAGTATCACAGACG	ACATTCCTGATGCCACCTT
**MKI67**	CGCCTGGTTACTATCAAAAGGA	GAAGCTGGATACGGATGTCA
**MLYC**	CTGCTGCGATCTTTTATTCCATC	CCAGGTATAGGTGACAGACTTG
**PDP1**	GGAAGAATCGTTTGGTCTCCT	CCATAGATCCTGCTCAGTTCAC
**PRKAA1**	AAGATCGGCCACTACATTCTG	ACAGCTACTTTATGCCCAGTC
**SDHB**	CAAGATTAAGAATGAAGTTGACTCTACT	AGAGTGTTGCCTCCATTGATG
**TFAM**	GCGCTCCCCCTTCAGTTTTG	GTTTTTGCATCTGGGTTCTGAGC
**NRF1**	GATGCTTCAGAATTGCCAACC	GTCATCTCACCTCCCTGTAAC
**18S rRNA**	GTAACCCGTTGAACCCCATT	GTCATCTCACCTCCCTGTAAC
**mt-tRNA Leu**	CACCCAAGAACAGGGTTTGT	TGGCCATGGGTATGTTGTTA

### Evaluation of mitochondrial DNA copy number

Mitochondrial DNA (mtDNA) copy number per diploid nuclear genome was determined by relative quantitative PCR (qPCR) as previously described (53). Total DNA from 5 x 10^6^ cells was extracted from cells using the gMAX DNA Mini Kit (IBI Scientific, Peosta, IA, USA) following manufacturer’s instruction. DNA was evaluated for the expression levels of mt-tRNA (Leu) and normalized to 18S rRNA using the 2^-ΔΔCT^ formula, where ΔCt = (Ct _nucDNA_ – Ct _mtDNA_) _(_ 52). Data was obtained using qPCR on a CFX Connect Real-Time PCR instrument (Bio-Rad) using the PerfeCTa^®^ SYBR^®^ Green SuperMix (Quantabio, Beverly, MA, USA) following the protocol: 95°C for 5 min along with 94°C for 15 sec, 52°C for 30 sec, and 72°C for 30 sec (x 39 cycles). Primer sequences used for mt-tRNA Leu analysis are presented in **Table 1**.

### Cell migration and invasion assays

To investigate the mobility and invasion of CLL cells, a total of 5 × 10^5^ cells/group was FBS-starved for 24 hours before the assay, mixed with 100 μL of media containing 2% FBS on the next day, and added to the insert chambers of a 24-well ThinCerts cell culture plate (8.0 μm pore size, ThermoFisher Scientific). For the cell invasion assay, a layer of Matrigel^®^ (0.5 mg/mL, Corning, Manassas, VA, USA) was added into the top transwell insert compartment to mimic the extracellular matrix. The lower plate wells were then filled with 750 μL of media containing 10% FBS. Following a 24-hour incubation at 37°C, non-migrating/non-invasive cells were removed and migrated/invaded CLLs were collected, stained with CD41 antibody for 15 min at 37°C, PBS washed and read with the flow cytometer.

### Bioinformatics analysis

Cytoscape version 3.8.1 (54) was used for network illustration. The interaction between genes was predicted by the plugin BisoGenet (55). It works by integrating the gene expression data with protein-protein interaction (PPI) data to create a comprehensive view of the interactions between genes and proteins in a given biological system using several different layout algorithms including force-directed, hierarchical, and circular layouts. CentiScaPe (Version 2.2) was applied for network topology. The list of genes involved in cancer metabolism was achieved from the DisGeNET tool (Version 7.0), a Bioinformatics tool for integrating and analyzing disorder-related genes in humans (56). DisGeNET uses databases like Comparative Toxicogenomics Database (CTD) (57), Genetic Association Database (GAD) (58), Mouse Genome Database (MGD) (59), and Online Mendelian Inheritance in Man (OMIM) (60) to identify human disease-related genes; and subsequently sorted them based on the number of sources, type of organism, and supported publications in the literature.

Expression analysis of target genes in human CLL samples was carried out using the public data sets GSE58211 (N = 287) and GSE40571 (N = 155) available on NCBI Gene Expression Omnibus (GEO, http://www.ncbi.nlm.nih.gov/geo/, accessed December 2021). Robust multi-array average (RMA) was used to remove the local biases and normalize the intensity expression of raw microarray samples to achieve the gene expression values. Hclust R-function performed hierarchical cluster analysis in R statistical environment to sort the normalized data. Identification of differentially expressed genes (DEGs) in sample files was carried out by linear models for microarray data (LIMMA) package. Annotation of genes was performed through the EnrichR server (http://amp.pharm.mssm.edu/EnrichR, accessed January 2022). The values were reported as significant if *p* < 0.05.

### Statistical analysis

Statistical analyses were performed with the SPSS software (Version 26, IBM Corporation). The normalization and homogeneity of variances of obtained data were done with the Shapiro-Wilk and Levene’s tests, respectively. Pairwise comparisons were made using an unpaired two-tailed Student t test. One/two-way analysis of variance (ANOVA) and Tukey’s multiple comparison tests were applied to find the statistically significant differences between multiple experimental groups as appropriate. The Pearson chi-square test was used to evaluate the distribution between categorical variables. The data are presented as the mean ± SEM (Standard Error of Mean) and deemed significant if *p* < 0.05.

## Results

### Platelet-derived microparticles interact with CLL cells

**Figure 1:**
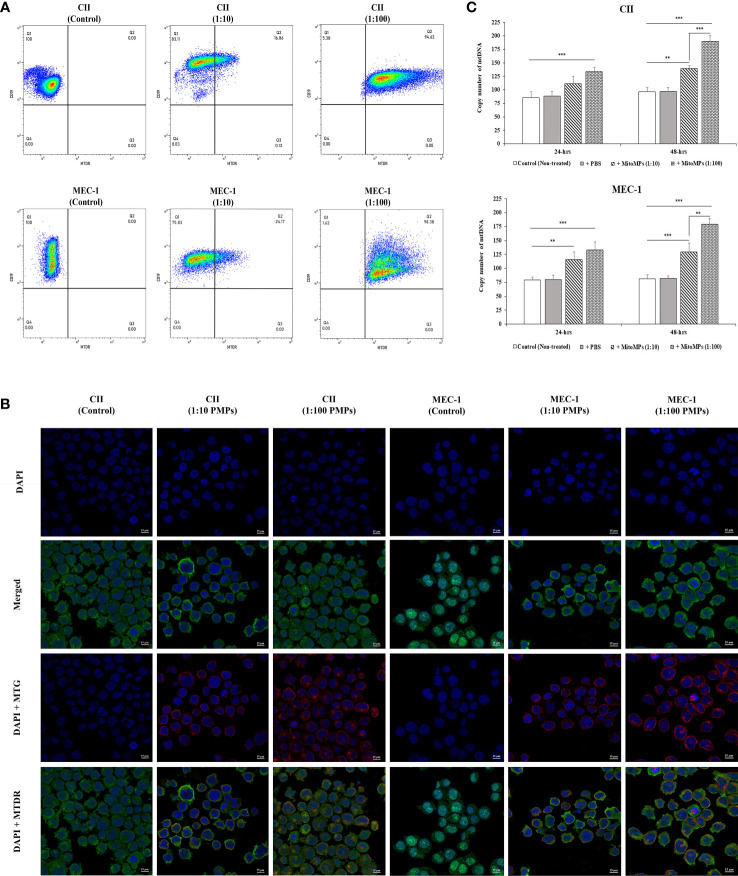
Interaction analysis of platelet-derived microparticles and CLL cells. **(A)** Flow cytometry was conducted for the characterization of CLL cell lines labeled with anti-human CD19 antibody either untreated (control) or co-incubated with platelet-derived microparticles (PMPs) labeled with MitoTracker Deep Red (MTDR). Co-incubations of CII (upper panels) or MEC-1 cells (lower panels) with PMPs were performed at 1:10 and 1:100 (cell/PMP) ratios for 48 hours. **(B)** CLL co-incubations (48 hours) with PMPs (1:10 and 1:100) were also photographed by fluorescence microscopy. CLL cell lines were labeled with DAPI and MitoTracker™ Green FM (MTG), whereas PMPs were labeled with MTDR. **(C)** Relative mitochondrial DNA copy number was obtained from cell/PMP co-cultures for 24 and 48 hours using Real-time PCR on leucine tRNA (mtDNA-tRNA-Leu) normalized with nuclear DNA copy number. Each value is the mean ± SEM of six separate experiments. The asterisk (*) indicates significant differences where **p < 0.01, and ***p < 0.001.

We and others have previously established PMP interaction and internalization into various types of recipient cancer cells (25, 61). To evaluate the potential PMP-mediated phenotypic regulation of leukemic cells, we first set out to validate the interaction of PMPs with CLL cell models using fluorescence-based approaches. Human leukemic cell lines (CII and MEC-1) were co-incubated with PMPs labeled with MitoTracker Deep Red (MTDR) at a ratio of 1:10 and 1:100 (cell:PMP) for 24 hours and examined for recipient cell acquired fluorescence transferred from labeled PMPs. Flow cytometry analysis demonstrated that both CLL cell lines acquired MTDR fluorescence indicating that CII and MEC-1 models acquire PMP-derived mitochondria (**Figure 1A**). Cell fluorescence from MTDR-labeled PMPs increased with cell/PMP ratios where nearly 100% of cells were positive for PMP-derived mitochondria at a 1:100 (cell/PMP) ratio after only 24 hours.

To further distinguish PMP internalization from outer cell surface interactions with target cells, we conducted fluorescence microcopy to depict intracellular compartmentalization of PMP-derived fluorescence. To do so, CII and MEC-1 cells were first stained with MitoTracker™ Green FM (MTG) and then co-incubated with MTDR-labeled PMPs at a ratio of 1:10 and 1:100 (cell: PMP) for 24 hours. Fluorescence microscopy confirmed that exogenous MTDR-labeled mitochondria provided by PMPs was transferred to CII and MEC-1 intracellular compartments. Interestingly, exogenous MTDR-labeled mitochondria also co-localized with the sub-compartmentalized region of native MTG-labeled mitochondria (**Figure 1B**).

To further validate horizontal transfer of platelet-derived mitochondria *via* PMPs, we performed qPCR analysis on mt-tRNA (Leu) as a mitochondrial DNA (mtDNA) or copy number indicator in CLL models co-incubated with PMPs. We observed that mtDNA was increased 1.39- and 1.91-fold in CII cells treated with 1:10 and 1:100 (cell/PMP) ratios for 48 hours, respectively (**Figure 1C**). Similar results were obtained for MEC-1 cells, which exhibited a 1.3- and 1.8-fold increase of mtDNA following treatments of PMPs with 1:10 and 1:100 ratios (48 h), respectively in comparison to control cells. Altogether, these results demonstrate that PMP cargo, notably mitochondria, are efficiently transferred into CLL cells, which result in the accumulation of mitochondrial content in recipient cells in a relatively short period of time (within 24 hours).

### PMPs impact CLL cell viability

**Figure 2:**
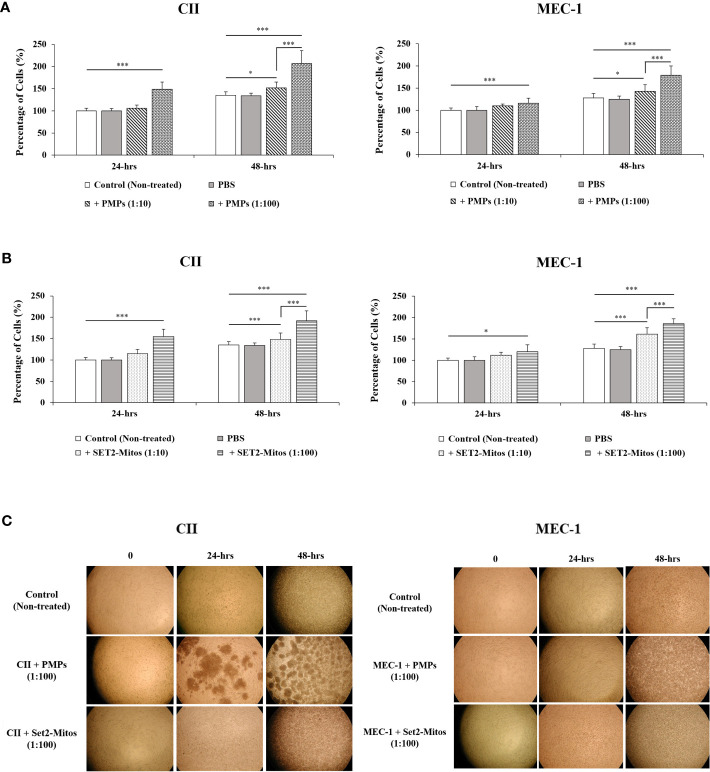
Impact of mitochondria acquisition on CLL viability. CLL cell lines were evaluated for cell viability at varying co-culture ratios (1:10 and 1:100) and incubation time (24 and 48 hours) with either **(A)** PMPs or **(B)** purified mitochondria isolated from the Set2 megakaryocytic cell line. Viability was established using a cell-based colorimetric assay (Cell Titer-Blue, Promega) combined a multi-well plate spectral analysis from a Synergy H1 Hybrid Multi-Mode Microplate Reader (Ex/Em: 560/590 nm). Each value is the mean ± SEM of six separate experiments. The asterisk (*) indicates significantly different (*p < 0.05, ***p < 0.001). **(C)** Light microscopy (40X) of CLLs co-incubations with PMPs or Set2-derived mitochondria was also performed for morphological examination.

Given the prominent role of mitochondria in cancer cell viability and growth, we next examined the phenotypic impact of PMP internalization on recipient CLL cancer processes. Cell viability was therefore assessed in CLL co-cultures with varying ratios of PMPs over time (24 and 48 hours) using a colorimetric cell-based assay. Accordingly, PMP co-culture with CII or MEC-1 cells induced significant cell growth, which was more pronounced after 48 hours. For example, CII and MEC-1 cells treated with PMPs (1:100) increased viability 1.9- and 1.7-fold, respectively, when compared to control cells (**Figure 2A**). Interestingly, raising the ratio of PMPs in CLL co-cultures (from 1:10 to 1:100) did not significantly enhance cell viability beyond the impact observed at a 1:10 ratio at 48 hours. These observations not only suggest that PMPs play a role in leukemia proliferation, but also show that a relatively small amount of PMP interactions is sufficient to induce significant CLL cell growth.

It is well established that PMP cargo encompasses a variety of bioactive content (beyond mitochondria) capable of provoking phenotypic changes in recipient target cells (22, 23). To discern the specific role of PMP mitochondria (from other bioactive cargo) in PMP-induced CLL cell growth (above), we treated CLL models with purified exogenous mitochondria organelles (**Figure 2A**). Technically, we made use of a platelet precursor cell line (i.e., Set2 megakaryocytes) to isolate and purify mitochondria organelles intended for CLL treatments. Here, CII and MEC-1 cells were co-cultured with different ratios (1:10 and 1:100) of purified Set2-derived mitochondria over time (24 and 48 hours) and confirmed previous observations where CLL cell viability was induced with increasing amounts of purified mitochondria. Specifically, treatments of CII and MEC-1 cells with Set2-derived mitochondria (1:100 ratio) increased viability (1.6- and 1.6-fold, respectively) after 48 hours (**Figure 2B**). Control experiments to validate the intracellular compartmentalization of Set2-derived mitochondria and levels of mtDNA in CLL recipient cells were also performed following mitochondria treatment using cell cytometry, fluorescence microscopy, and qPCR (**Supplementary Figures 1A-C**, respectively). Morphological analysis of CLL co-cultures (1:100 ratio) at various time points was also conducted by light microscopy (**Figure 2C**). Images of co-cultured cells show typical cell agglomeration, which is characteristic of cell growth.

Meanwhile, to better relate the direct role of mitochondria enhancement on cells proliferation, we also examined the viability of CLL cells in the presence of PMPs containing the damaged/disrupted mitochondria (**Supplementary Figure 2**). We used two previously proven approaches where the first one consisted of freeze-thawing the PMPs prior to the co-incubation with CLLs (62). Since mitochondria are sensitive to temperature changes, cycles of freezing and thawing causes damage to their function thus impairing the motility, plasma membrane integrity and mitochondria function of boar spermatozoa through generating excessive ROS (62). The second strategy was treating the PMPs with ROT/AmA compounds, as the mitochondrial respiratory chain complex I and III inhibitors, before co-incubation with the CLL cell groups (63, 64). Upon co-incubation of leukemic cells with frozen-thawed PMPs, we observed no significant impact on cell viability in comparison to non-treated control cells (**Supplementary Figure 2A**). Similar observations were noted PMPs treated with ROT/AmA prior to co-incubation with CLL cells (**Supplementary Figure 2B**). These results showed that the proliferative impact of PMPs on cancer cells markedly depends on the health status of PMPs mitochondria. Altogether, these findings support an important contribution for PMPs in CLL proliferation. Our results also suggest that the mitochondrial component of PMP cargo is primarily responsible for accelerated growth rates of CLL cells following PMP internalization.

### PMPs impact leukemic cell bioenergetic states

**Figure 3:**
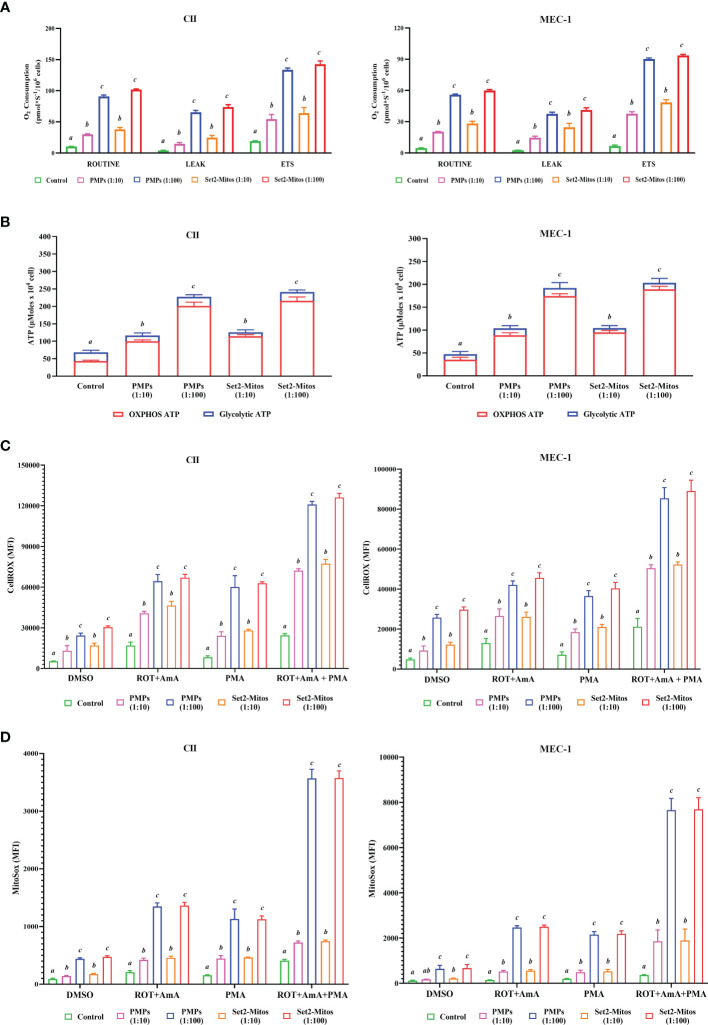
Impact of PMPs on CLL metabolic functions. CLL cell lines (CII and MEC-1) were evaluated for metabolic functions at varying co-culture ratios (1:10 and 1:100) and incubation periods (24 and 48 hours) with either PMPs or purified mitochondria isolated from the Set2 megakaryocytic cell line. **(A)** Oxygen consumption rate (OCR) was determined using an Oroboros-O2k for: ROUTINE respiration as the basal state; the non-phosphorylating resting state (LEAK respiration); and the electron transport system (ETS) capacity. **(B)** Total ATP and glycolytic ATP levels were evaluated using a firefly luciferase-based assay. Relative ATP concentrations related to specific pathways (oxidative phosphorylation/OXPHOS versus glycolysis) were determined by cell treatments to inhibit mitochondrial complexes I and III using Rotenone (ROT) and Antimycin A (AmA), respectively. **(C, D)** Cells were treated with AmA, ROT, and phorbol 12-myristate 13-acetate (PMA) as ROS inducers. Thereafter, samples were mixed with **(C)** CellROX green reagent and SYTOX Red Dead Cell Stain prior analysis by flow cytometry at Ex/Em: 508/525 nm (CellROX green) and 640/658 nm (SYTOX Red Dead Cell Stain) to determine intracellular total ROS content; or with **(D)** MitoSOX™ reagent prior analysis by flow cytometry at Ex/Em: 510/580 nm to determine mitochondrial superoxide content. N-acetylcysteine (NAC) and Tert-butyl hydroperoxide (TBHP) were used as negative and positive controls, respectively. Results are expressed as the mean ± SEM of six biological experiments. One-way ANOVA followed by Tukey’s multiple comparisons test show significant differences in the values presenting different superscript letters (*p* < 0.05).

Mitochondrial transfer (or “mito-transfer”) is a well described phenomenon amongst cell types and has been shown to modulate recipient cell metabolic function (65–67). Since metabolic plasticity represents a hallmark of cancer progression, we investigated the impact of PMP internalization, or Set2-purified mitochondria, on several mitochondrial functions of our human leukemic cell models. First, co-cultures consisting of CLL lines (CII and MEC-1) with either PMPs or Set2-isolated mitochondria, were examined for cellular respiration through OCR using a high-resolution respirometer. We found that CLL co-cultures with PMPs (1:10) significantly increased ROUTINE (basal) respiration in both CII and MEC-1 cells (2.9- and 4.4-fold, respectively) after 48 hours when compared to controls samples (**Figure 3A**). These levels were even more pronounced (~2.7-fold higher for both cell lines) in 1:100 culture ratios (**Figure 3A**). Interestingly, co-cultures with Set2-isolated mitochondria yielded identical increases in cell ROUTINE respiration in comparison to PMP-treated cells (**Figure 3A**). These results suggest that PMP-induced basal respiration state in CLL cells is likely dependent on the transfer and function of PMP mitochondria.

We also observed that levels of non-phosphorylating resting LEAK state, after blocking the ATP synthesis with oligomycin, were also increased in PMP-treated CLL cultures, which were further accentuated with 1:100 ratios for both CII and MEC-1 cells (15.1- and 14.2-fold, respectively; **Figure 3A**). These increments were reciprocated in cultures treated with Set2-purified mitochondria under the same conditions (15.9- and 17.2-fold, respectively; **Figure 3A**). Finally, uncoupled respiratory capacity determined through the maximal capacity of the mitochondrial electron transport system (ETS), was also increased in PMP co-cultures, which was further accentuated in 1:100 culture ratios of CII and MEC-1 cells (7- and 13.4-fold, respectively) over control samples (**Figure 3A**). Likewise, Set2-isolated mitochondria co-cultures mimicked these increases in CII and MEC-1 cells (7.5- and 13.8-fold, respectively) in comparison to their related-control groups (**Figure 3A**).

To further elucidate the impact of PMPs on CLL mitochondrial-dependent functions, we examined ATP levels using bioluminescence assays. Total ATP levels in CII cells were increased 1.7- and 3.3-fold when cultured with PMPs (1:10 and 1:100, respectively) (**Figure 3B**). Similar ATP level increases in MEC-1 cells were also observed (2.2- and 4.1-fold) following 1:10 and 1:100 culturing ratios with PMPs, respectively (**Figure 3B**). Further investigation into the mechanistic source responsible for induced ATP levels, revealed that the OXPHOS pathway was mainly responsible (**Figure 3B**). In control experiments using Set2-derived mitochondria treatments, we observed comparable induced ATP levels suggesting that PMP-induced ATP levels in CLL cells are likely dependent on the transfer and function of PMP mitochondria.

Next, we examined intracellular ROS and mitochondrial superoxide levels in PMP-recipient leukemic cells treated with either rotenone (ROT, inhibitor of complex I), antimycin A (AmA, inhibitor of complex III), or phorbol 12-myristate 13-acetate (PMA, ROS inducer). The data depicted a significant increase in total ROS in CII cells co-cultured with PMPs (1:10 and 1:100) of 2.0- and 4.6-fold, respectively in comparison to total intracellular ROS levels from control cells alone (**Figure 3C**). To further discern the contribution of mitochondria-derived ROS production, mitochondrial superoxide was analyzed and show 1.6- and 4.8-fold increases in CII cells co-cultured with PMPs (1:10 and 1:100, respectively) when compared to their respective controls (**Figure 3D**). Similarity, MEC-1 treated with PMPs (1:10 and 1:100) induced 1.7- and 5.3-fold increments respectively, of total intracellular ROS (**Figure 3C**), where mitochondrial superoxide was increased 1.6- and 5.7-fold respectively (**Figure 3D**).

Given that Complex I is responsible for the transfer of electrons from NADH to ubiquinone (coenzyme Q) (68, 69) and that Complex III is responsible for the transfer of electrons from ubiquinone to cytochrome C (68), the combinatory use of inhibitors ROT and AmA is generally used to increase ROS levels (69). We therefore treated our cells with a ROT+AmA combination, which raised intracellular and mitochondrial superoxide levels by 3.8- and 6.4-fold in CII cells (1:100), respectively (**Figures 3C, D**). In comparison, MEC-1 intracellular ROS and mitochondrial superoxide were 3.2- and 17.2-fold higher following PMP treatments (**Figure 3C, D**). Upon ROS stimulation using PMA treatment (70–72), CII cells co-cultured with PMPs (1:100) enhanced intracellular ROS and mitochondria superoxide levels by 7.2- and 7.5-fold, respectively (**Figures 3C, D**). Likewise, PMP treated MEC-1 cells induced levels 5.1-fold (intracellular ROS) and 10.7-fold (mitochondrial superoxide), in comparison to non-treated leukemic cells (**Figures 3C, D**).

The maximum potential of ROS generation between study models was tested with mixtures of ROT+AmA+PMA. Intracellular ROS levels in CII cells treated with PMPs (1:100) increased 4.9-fold, whereas mitochondrial superoxide was raised 8.8-fold in comparison to control samples (**Figures 3C, D**). On the other hand, MEC-1 cells under these conditions raised intracellular ROS levels 4.0-fold, whereas mitochondrial superoxide increased 21.0-fold when compared to non-treated cells (**Figures 3C, D**).

Throughout these experiments, we also made used of Set2-derived mitochondria controls in parallel. We found that purified mitochondria treatments induced comparable levels of both intracellular ROS and mitochondrial superoxide as observed in PMP treatments (**Figures 3C, D**). These observations therefore suggest that total ROS production in CLL cells is likely dependent on the transfer of mitochondrial content and functions provided by PMPs. Overall, our data indicates a statistically significant increase in ROS levels, which may be attributable to either increased ROS production, or decreased antioxidant capacity of CLL cells following PMPs interaction. These results strongly suggest that PMPs enable CLL cells to gain mitochondrial-dependent functions supporting metabolic reprogramming and plasticity, which are essential for cancer malignancy.

### CLL disease correlates with heightened core metabolic-related gene expression profiles

**Figure 4:**
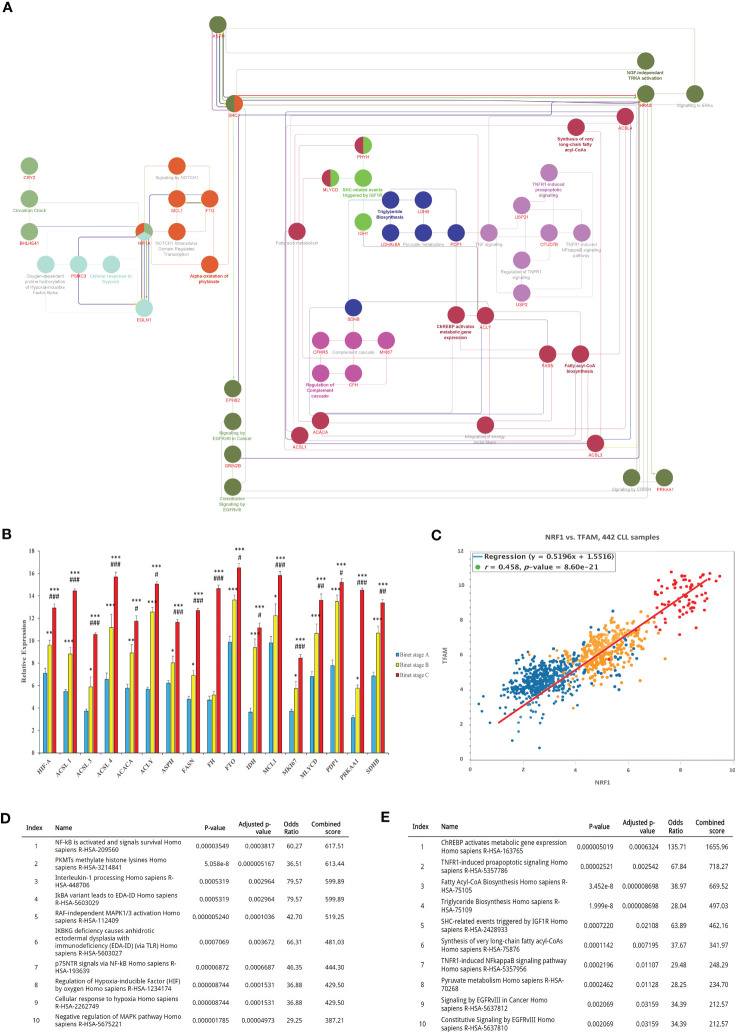
Bioinformatics identification and analysis of core metabolic-related genes involved in CLL progression. **(A)** Identification of key genes involved in CLL metabolism. The interaction between genes was visualized by the Cytoscape plugins BisoGenet and CentiScaPe, and subsequently annotated using the EnrichR. **(B)** Expression analysis of the most differentially expressed metabolic-related genes (DEGs) between CLL datasets were then calculated and plotted in relative expression. **(C)** Pearson Chi-square test of CLL samples was then analyzed to plot the expression levels of TFAM and NRF1 as mitochondrial biogenesis indicators in relation to Binet stages of clinical samples. **(D)** The most active signaling pathways from patients with early onset of CLL (Binet stage A), or **(E)** advanced disease (CLL Binet stage C) were also identified and listed. Each value is the mean ± SEM of six separate experiments. The asterisk (*) and number sign (#) indicate significantly different from the control group (non-PMP treated), and the 1:10 PMP-treated group, respectively (*p* <.05).

Given the benefits from the acquisition of metabolic content and function in cancer cells, we made use of bioinformatics analyses to identify the mechanisms involved in metabolic reprogramming during disease progression and the PMP treatment. Using the BisoGenet tool, a complex network of metabolic-related genes actively involved in CLL development was developed. Network genes were then filtered based on their betweenness centrality and streamed into a smaller network consisting of 160 high-degree nodes (Hubs) where 525 edges were created as potentially key players in cancer metabolism (**Supplementary Figure 3**). The network was then used to identify the most potential pathways encompassing these genes and highlighted: ChREBP activates metabolic gene expression (R-HSA-163765) and, the Fatty acyl-CoA biosynthesis (R-HSA-75105), as the most impacted signaling cascades in CLLs (**Figure 4A**). The expression levels of genes associated to these identified pathways were then applied to a cohort of CLL patients (n=442) based on their corresponding Binet staging (i.e., stage A (n)=184, stage B (n)=179, and stage C (n)=79) to define the most significant modulated genes associated with CLL disease progression. The average expression values of the profiled gene lists per patient are provided in **Supplementary File S4**. Through our analysis of CLL samples, we first identified an expression pattern from 17 metabolic-related genes significantly altered through disease progression (i.e., Binet stages A to C). These genes include: Hypoxia-inducible factor-alpha (HIF-A); ATP citrate lyase (ACLY); Long-chain fatty acyl-CoA ligase (ACSL1, 3 and 4); Pyruvate dehydrogenase (PDH); Fatty acid synthase (FASN); Acetyl-CoA carboxylase (ACACA); Malonyl-CoA decarboxylase (MCD); Isocitrate dehydrogenase (IDH); Fumarate hydratase (FH); Aspartate Beta-Hydroxylase (ASPH); Alpha-ketoglutarate-dependent dioxygenase (FTO); Myeloid cell leukemia-1 (MCL1); Malonyl-CoA Decarboxylase (MLYCD); Pyruvate dehydrogenase phosphatase (PDP1); and, Protein Kinase AMP-Activated Catalytic Subunit Alpha 1 (PRKAA1) (**Figure 4B**). These genes were then considered as metabolic reprogramming indicators for future experiments. In line with these findings, we also observed that expression levels of Transcription factor A mitochondrial (TFAM) and the Nuclear respiratory factor 1 (NRF1), two prominent factors involved in mitochondrial biogenesis, were concomitantly increased with CLL staging (**Figure 4C**). These results not only demonstrate that CLL progression directly correlates with rising mitochondrial content in cells; but also, with the levels of metabolic-gene expression.

Using our enrichment analysis, we then categorically sorted our lists of modulated gene expressions, along with their associated pathways, to reveal the most active signaling cascades upon the onset of CLL development (Binet stage A). For example, NF-κB and survival signals (R-HSA-209560); PKMTs methylate histone lysines (R-HSA-3214841); and Interleukin-1 processing (R-HSA-448706) were all highly activated in early stages of CLL disease (**Figure 4D**). On the other hand, signaling pathways associated with advanced disease stages (Binet stage C) identified the pathways of ChREBP activates metabolic gene expression (R-HSA-163765), TNFR1-induced proapoptotic signaling (R-HSA-5357786), and Fatty Acyl-CoA Biosynthesis (R-HSA-75105), all which mitochondria plays a central role in advanced CLL disease (Binet stage C) (**Figure 4E**). These findings indicate that metabolic gene expression supporting metabolic reprogramming are tightly correlated to CLL progression and suggest that these gene profiles are likely impactful regulators of this process.

### PMPs regulate metabolic reprogramming and protection of CLL cells

**Figure 5:**
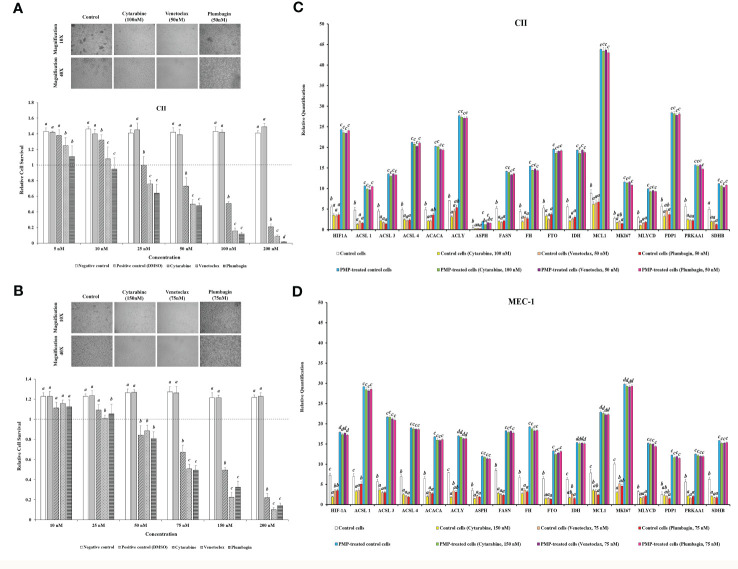
Impact of PMPs on CLL chemoresistance and metabolic gene expression. **(A)** CII and **(B)** MEC-1 cell lines were co-incubated with PMPs (1:100, 48 h) and then treated with increasing amounts of either Cytarabine, Venetoclax, and Plumbagin (5-200 nM) for 24 hours to establish the lethal doses for 50% mortality (LD50). Control groups were treated with DMSO as dilution buffer. Light microscopy of cells treated at their respective LD50 are also presented for morphological analysis. **(C, D)** Real-time PCR analysis was performed to evaluate differentially expressed metabolic-related genes (DEGs) in CLL groups in the presence of chemo-drugs where each column represents the PMP-treated/non-treated cell ratio. Control groups were treated with DMSO as dilution buffer. Results are expressed as the mean ± SEM of six biological experiments. One-way ANOVA followed by Tukey’s multiple comparisons test show significant differences in the values presenting different superscript letters (*p* < 0.05).

Based on our results showing that PMPs enable CLL metabolic reprogramming, we investigated the potential impact of PMPs on metabolic transition as part of CLL development and progression. For this purpose, CLL cells were co-cultured with PMPs (1:100) and tested for changes in metabolic-genes expression (identified in **Figure 4**) associated with disease progression. These experiments were also combined with treatments of common anti-leukemia drugs (i.e., Cytarabine, Venetoclax, and Plumbagin) as these compounds elicit well-known mitochondrial inhibitory features. First, we established the median lethal dose (LD50) of anti-cancer drugs for both CII and MEC-1 cell lines as follows: ~100 nM and ~150 nM, respectively, for Cytarabine; ~50 nM and ~75 nM, respectively, for Venetoclax; and ~50 nM and ~ 75 nM, respectively, for Plumbagin (**Figures 5A, B**). The correlation between cell viability and metabolic-reprogramming in PMP-recipient CLL cells was then investigated using RT-qPCR to measure the relative expression levels from our panel of metabolic gene. We observed that PMP treatment of CLL cells induced the expression levels from all metabolic genes associated with advanced disease progression in both CII (**Figure 5C**) and MEC-1 (**Figure 5D**) cells with or without anti-cancer drug (LD50) treatments. For example, PMP treatment induced the expression of HIF-1A, which is known to promote the interaction between neoplastic B cells and their microenvironments (73), induces the generation of tumor-associated macrophages (TAM) (74), and participate in drug-resistance (75). HIF-1A expression was increased 4.3-fold in PMP-recipient CII cells compared to non-treated cells alone. However, this difference was increased in the presence of Cytarabine (6.9-fold), Venetoclax (7.4-fold), and Plumbagin (6.7-fold). This data demonstrates that PMPs increase the expression profiles of metabolic genes associated with CLL progression.

We further repeated this assay with CLLs co-incubated with (1:100) isolated Set2-Mitos in order to evaluate the role of mitochondria acquisition in the observed metabolic reprogramming (**Supplementary Figure 5**). Similar to PMP-recipient cell groups, our observations indicated a statistically significant improvement in metabolic gene expression patterns in response to Set2 mitochondria internalization into the CLL cells. Meanwhile, this expression pattern didn’t markedly change in the presence of the determined LD50 concentration of Cytarabine, Venetoclax, and Plumbagin for CII and MEC-1 cells. This data demonstrates that the observed alteration in metabolic gene expression patterns of CLLs co-incubated with PMPs mostly derived from mitochondrial content increment.

### PMPs impact CLL cell-cycle progression and apoptosis

Reports have previously characterized CLL cells with substantially greater levels of mitochondrial mass and function when compared to healthy B-cells (11, 15). Given our observations of PMP-induced expression of metabolic genes associated with CLL disease progression, we set out to examine if this differential display in metabolic gene expression translates into altered cancer cell phenotypes associated with CLL progression. We therefore co-cultured CLL cells with PMPs (1:100 for 48 hours) and examined cell viability, growth, and programmed cell death (apoptosis) in response to anti-cancer drug treatments (LD50 for 24 hours) using Annexin-V/PI labeling. The stained cells were then analyzed by flow cytometer and sorted into four distinctive quarter zones (Q) including necrotic cells or Q1 (Anx^-^/PI^+^), late apoptotic or Q2 (Anx^+^/PI^+^), early apoptotic cells or Q3 (Anx^+^/PI^-^), and viable cells or Q4 (Anx^-^/PI^-^).

**Figure 6:**
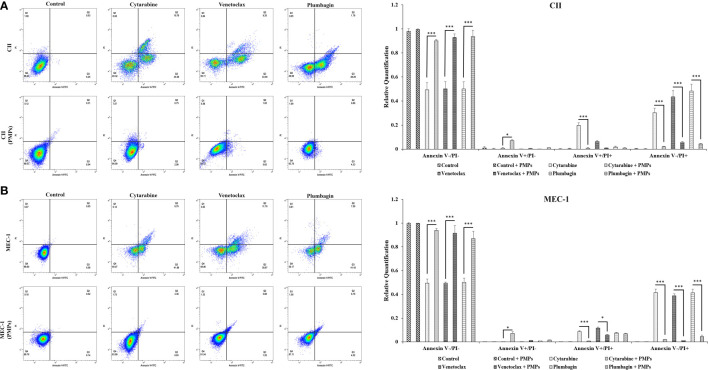
Effects of PMPs on CLL survival and apoptosis. CLL cells were co-incubated with PMPs (1:100, 48 h) prior to treatments with the cells’ respective LD50 of Cytarabine, Venetoclax, and Plumbagin for 24 hours for the evaluation of drug cytotoxic events. Flow cytometry analysis in **(A)** CII and **(B)** MEC-1 cells stained with Propidium Iodide (PI, X‐axis) and Annexin‐FITC (X‐axis) sorted cell populations according to distinctive quarter zones (Q), including the large nuclear fragments/debris or Q1 (Anx^-^/PI^+^), necrosis cells or Q2 (Anx^+^/PI^+^), apoptotic cells or Q3 (Anx^+^/PI^-^), and the living cells or Q4 (Anx^-^/PI^-^). Values for each quadrant were then plotted in relation to their respective control groups (DMSO). Each value is the mean ± SEM of six separate experiments. The asterisk (*) indicates significantly different (*p < 0.05, ***p < 0.001).

As expected, anti-cancer drug treatments induced apoptosis in both CLL models. For example, drug treatments induced early apoptosis (Q3) to 30.3% (Cytarabine), 43.5% (Venetoclax), and 48.2% (Plumbagin) in CII cells (**Figure 6A**). Meanwhile, drug treatments in MEC-1 cells induced early apoptotic events (Q3) by 41.6% (Cytarabine), 38.9% (Venetoclax), and 41.4% (Plumbagin) under the same conditions (**Figure 6B**). However, upon co-culturing conditions with PMPs (1:100, 48 hours), cells were completely refractory to drug toxicity. For example, CII cells treated with PMPs underwent early apoptosis (Q3) in only 2.2% (Cytarabine), 5.65% (Venetoclax), and 4.33% (Plumbagin) in comparison to drug-treated control cells (**Figure 6A**). This represents an inhibition of approximately 93.2% (Cytarabine), 87.1% (Venetoclax), and 91.1% (Plumbagin) in comparison to drug-treated control cells (without PMPs). Similar results were also obtained in MEC-1 co-cultured with PMPs where early apoptosis was inhibited by 97.8% (Cytarabine), 96.8% (Venetoclax), and 89.1% (Plumbagin) in comparison to drug-treated controls (**Figure 6B**). PMP treatments of CLL cells also noticeably increased the viable (Q4) population by 1.89-fold (Cytarabine), 1.85-fold (Venetoclax), and 1.74-fold (Plumbagin) when compared to the non-PMP-treated control cells (**Figure 6A**). Likewise, MEC-1 co-cultures with PMPs increased viability by 1.83-fold (Cytarabine), 1.85-fold (Venetoclax), and 1.87-fold (Plumbagin) when compared to drug treatments in cells alone (**Figure 6B**). These results demonstrate that PMP-treated CLL cells are more resistant to conventional anti-cancer drug toxicity.

To have a better conclusion, this test was also repeated with CLLs co-incubated with (1:100) isolated Set2-Mitos (**Supplementary Figure 6**). A similar observation has been achieved through flow cytometry assessment of cell proportions in apoptosis when leukemic cells received isolated mitochondria prior to drug treatment. Collectively, this data indicates that mitochondria acquisition in CLL cells has a major impact on cancer cells performance against the therapeutic agents.

**Figure 7:**
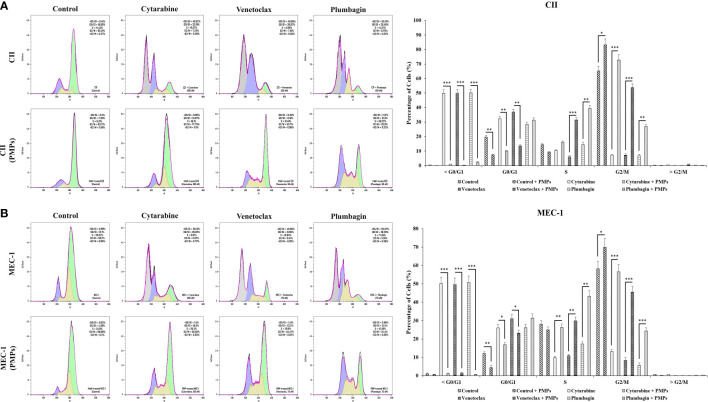
Impact of PMPs on CLL cycle progression. CLL cells were co-incubated with PMPs (1:100, 48 h) prior to treatments with the cells’ respective LD50 of Cytarabine, Venetoclax, and Plumbagin for 24 hours then evaluated for cycle analysis using flow cytometry. Cell accumulation in each cell cycle phase (i.e., <G0/G1, G0/G1, S, G2/M, and >G2/M) for **(A)** CII and **(B)** MEC-1 cells stained with Propidium Iodide and RNase A was then plotted in relation to control groups treated with DMSO. Each value is the mean ± SEM of six separate experiments. The asterisk (*) indicates significantly different (*p < 0.05, **p < 0.01, ***p < 0.001).

To provide insight into the regulation of CLL cycling processes modulated by PMPs, we performed cell-cycle distribution analysis on CLL models treated (or not) with anti-cancer drugs. Our results first show that the basal proportion of the PMP-recipient CLL cells in G2/M (dividing and growing cells) phase was about 1.27-fold in CII (**Figure 7A**) and 1.2-fold higher in MEC-1 (**Figure 7B**) when compared to non-treated control cells. These observations demonstrate that PMPs promote CLL cell cycling. Cell cycle analysis also confirmed our previous observations where toxic drug treatments of CLL cells led to an accumulation of cells in sub-G1 (<G0/G1), a phase representing cellular debris, late apoptotic, and necrotic cells. However, PMP treatments of CLL cells completely inhibited sub-G1 (<G0/G1) accumulation of both CII (**Figure 7A**) and MEC-1 (**Figure 7B**) cell models treated with anti-cancer drugs.

Furthermore, when CLL cells were co-cultured with PMPs prior to anti-cancer drug treatments, cell accumulation in G2/M was increased 10.1-fold (Cytarabine), 7.5-fold (Venetoclax), and 4.0-fold (Plumbagin) for CII cells (**Figure 7A**). Similar observations were noted in MEC-1 cells (**Figure 7B**) where cell accumulation in G2/M phase was enhanced 4.3-fold (Cytarabine), 5.4-fold (Venetoclax), and 4.4-fold (Plumbagin) when treated with PMPs). These results demonstrate that PMPs promote CLL growth and provide anticancer drug resistance.

In addition to the latter results, cell cycle analysis of CLLs incubated with mitochondria (1:100) isolated from Set2 cells also showed a statistically decrement in cells number accumulated in >G0/G1 following treatment with the LD50 dose of Cytarabine, Venetoclax, and Plumbagin chemo-drugs (**Supplementary Figure 7**). The data supports the notion that mitochondria acquisition *via* PMPs in CLL cells lowers sensitivity to standard leukemic treatments.

### PMPs promote CLL migration and invasion properties

**Figure 8:**
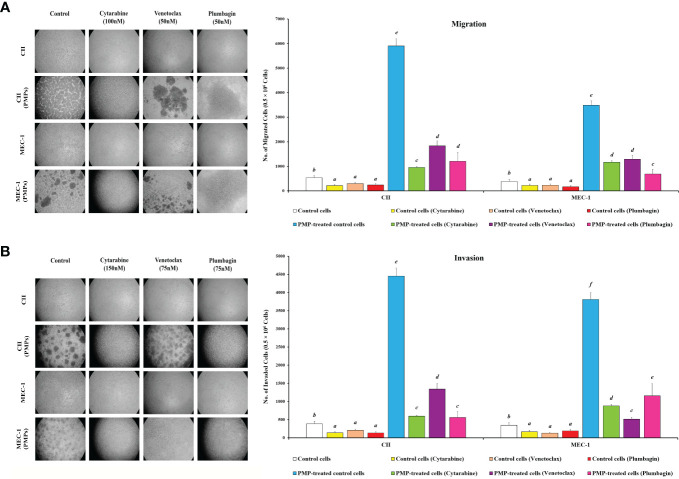
Effect of PMPs on CLL malignant processes. CLL cells were co-incubated with PMPs (1:100, 48 h) prior to treatments with the cells’ respective LD50 of Cytarabine, Venetoclax, and Plumbagin for 24 hours then evaluated for **(A)** migration and **(B)** invasion capacity using 24-well ThinCerts. Control groups were treated with DMSO as dilution buffer. Light microscopy of invading cells treated at their respective LD50 are also presented for morphological analysis (left panels). Results are expressed as the mean ± SEM of 6 biological experiments. One-way ANOVA followed by Tukey’s multiple comparisons test show significant differences in the values presenting different superscript letters (*p* < 0.05).

Given our observations that PMPs induce CLL aggressivity through enhanced growth and drug resistance, we wanted to determine the impact of PMPs on other CLL malignant processes such as migration and invasion properties. First, migration assays were performed and indicated that the mobility of leukemic cells treated with PMPs (1:100, 48 hours) was significantly increased when compared control samples without PMPs. Specifically, relative migration of CLL co-cultures was increased by 11.1- and 9.1-fold in CII and MEC-1, respectively (**Figure 8A**). We next evaluated migration properties in the presence of anti-cancer drug treatments. We found that the migration capacity of CLL cells co-cultured with PMPs was significantly improved following drug treatments with Cytarabine (4.5-fold), Venetoclax (6.2-fold), and Plumbagin (5.0-fold) (**Figure 8A**). Similar results were also obtained from MEC-1 cells where PMPs increased migration by 5.2-fold (Cytarabine), 5.5-fold (Venetoclax), and 4.0-fold (Plumbagin) when compared to drug treated control cells (**Figure 8A**).

To evaluate invasion potential, we made use of cell culture inserts layered with Matrigel^®^ mixture. We found that basal invasion capacity of CLL cells co-culture with PMPs was significantly increased by 11.6- and 10.9-fold in CII and MEC-1 cells, respectively when compared to non-treated CLL cells (**Figure 8B**). These trends were also maintained following drug treatments where PMPs significantly increased the invasion capacity 4.3-fold (Cytarabine), 4.1-fold (Venetoclax), and 6.4-fold (Plumbagin) of CII cells. Equally, MEC-1 cells co-cultured with PMPs also exhibited increased invasion capacity following drug treatments about 5.2-fold (Cytarabine), 4.0-fold (Venetoclax), and 6.0-fold (Plumbagin) higher than their drug-treated control cells (**Figure 8B**). Together, these results demonstrate that PMPs promote CLL invasive features (i.e., migration and invasion) even in the presence of anti-cancer drugs.

To evaluate the role of mitochondria in these events, we performed migration/invasion analysis on CLLs incubated with Set2-derived mitochondria (1:100). We observed a significant increase of mobility (**Supplementary Figure 8A**) and invasiveness (**Supplementary Figure 8B**) following mitochondria acquisition even after treating with an LD50 dose of chemo-drugs including Cytarabine, Venetoclax, and Plumbagin in comparison to control cells. These observations highlight the importance of mitochondrial function in cancer cell resistance to anticancer drugs. Together, these results strongly suggest an impactful role for PMPs in enhanced CLL malignancy, anti-cancer drug resistance, and disease progression.

## Discussion

Platelets contain various molecules that have been linked to cancer progression, including growth factors like platelet-derived growth factor (PDGF) and transforming growth factor-beta (TGF-β), cytokines such as interleukin-1 beta (IL-1β) and interleukin-6 (IL-6), chemokines like CXCL12 (SDF-1), and extracellular matrix proteins like fibronectin and vitronectin (76–78). These molecules can promote tumor cell proliferation, survival, angiogenesis, invasion, and metastasis. However, the exact roles of platelet cargos in cancer development and progression are still not fully understood and may depend on tumor type and stage (76–78). Further research is needed to elucidate the specific mechanisms involved and develop new therapeutic strategies targeting platelet-associated molecules in cancer.

It is well established that inflammation is a critical component of cancer progression (reviewed in (16)). This is due to the array of signaling mediators dictating cancer malignant processes leading to disease progression. Platelets are particularly active in these events, notably through their production of PMPs, which are reported as prominent modulators of cancer processes and progression (35, 79, 80). For example, PMPs have demonstrated a role in carcinogenesis through the direct transfer of their bioactive cargo and phenotypic trait acquisition such as invasive capacity and chemoresistance in recipient cancer cells (36, 81–83). Accordingly, we have previously reported a new subpopulation of PMPs, which encompasses functional mitochondria thus adding a new layer of complexity to the phenotypic impact in recipient cells upon PMP internalization (25, 32). Given that mitochondrial functions are an integral part of cancer processes such as cell growth, energy transduction, redox regulation, invasive capacity, and drug resistance (43–47), PMP-mediated transfer of mitochondrial content and functions represents a significant phenomenon supporting cancer progression. In this study, we report the first evidence of mitochondrial transfer (mito-transfer) in leukemia cells *via* PMPs. These events result in increased levels of mitochondrial content and functions, which are accompanied by metabolic reprogramming and phenotypic changes supporting CLL malignant processes essential in disease progression.

Fundamentally, metabolic adaptation is crucial for the survival of cancer cells in various microenvironments. Cancer metabolism is not simply the enhancement of a particular process, but rather a metabolic plasticity enabling cancer cells to consistently modify their metabolism to adapt to altering environmental challenges (9). This metabolic plasticity, which is generally absent in differentiated healthy cells, is a hallmark of cancer cell resilience. Accordingly, this is well demonstrated in our profiling of metabolic-gene expression patterns in CLL patients at various stages of disease (**Figure 4**). Our results demonstrate that CLL disease progression not only directly correlates with levels of metabolic-gene expression, but also with the amounts of mitochondrial mass in cancer cells. These observations are consistent with others reporting that mitochondrial mass and functions are intimately linked to CLL disease progression (11–15). Our results also corroborate previous studies demonstrating that CLL cells possess greater mitochondrial mass, mtDNA copy number, and functions when compared to healthy B-cells (11, 15). We further validate the relationship between mitochondria acquisition and malignant features by demonstrating enhanced CLL proliferation, drug resistance, and migration/invasion capacities following PMP co-cultures or treatments with purified mitochondria. Evidently, the acquisition of mitochondrial mass in CLL reflects the cells’ extensive demand in metabolic versatility supporting phenotypic adaptation during disease progression. Fundamentally, based on the effectiveness of PMPs to increase CLL mitochondrial mass and functions (**Figures 1**, **3**), which concomitantly leads to metabolic-gene expression and malignant CLL processes (**Figure 5**), PMPs solely could govern CLL disease progression. Besides, previous protein characterization of PMPs has shown that these vesicles cargo different types of proteins involved in various signaling processes (24). A superficial comparison analysis of this list with the metabolic pathways retrieved from our Bioinformatics data revealed that PMP proteins like ACLY, CD36, FASN, GNAI2, GNAQ, GNB2, GNG5, ITPR1, PPP2CA, PPP2R1A, PRKACB, PRKAR1A, PRKCA, SLC2A1 and TKT might also be involved in the metabolic reprogramming through the modulation of metabolic pathways like “ChREBP activates metabolic gene expression (R-HSA-163765)” depicted in our report. These events are substantially more impactful when considering CLL patients have increased levels of PMPs in their circulation (84). Future *in vivo* experimentation using animal models (ex: Eμ-TCL1 (85, 86)) should provide a better understanding of PMP impact on CLL cancer biology and disease progression.

Deciphering the regulatory networks of metabolic reprograming supporting cancer metastasis is extremely relevant and reflected by its attention for therapeutic targeting (46, 47, 87). PMPs are particularly noteworthy as cancer metabolic modulators as they harness multiple hallmark features enabling cancer progression (6). On one hand, PMPs package inflammatory components from their parental platelet cells, which are known to provide inflammation and signaling mediators driving malignant cancer processes (16, 18–20). On the other hand, PMPs also package functional mitochondria, which we demonstrate to foster metabolic reprogramming and cancer processes in recipient cancer cells. Although our characterization of PMP-mediated transfer of mitochondrial content and functions into cancer cells is novel, others have previously described mito-transfer phenomena mediated through extracellular vesicles from other cell types (66, 88–90). However, these findings were shown to be physiologically confined to neural and glia cell activation processes. Mito-transfer events have also been described using other routes of allocation such as tunneling nanotubes (TNT), Cx43 gap junctions, and transplantation, all of which result in recipient cell metabolic changes (65–67). Nonetheless, given that platelets represent the largest source of microparticles in the human body, PMPs therefore represent a significant means for mitochondrial acquisition into permissive cancer cells (27, 28, 91, 92).

Metabolic-related pathways in eukaryotic cells are directly affected by the number and functions of their “powerhouse” mitochondria; however, the exact impact of mitochondrial acquisition on cancer cell signaling cascades has not been fully elucidated. A study from Tan et al. (2015) has shown that the acquisition of stromal cell-derived mitochondria by mitochondria-negative (i.e., rho zero, or ρ0) cancer cells, restores cell growth and tumorigenicity *in vivo* through the activation of OXPHOS (93). In leukemia, mitochondria acquisition has been previously described in hematological lesions such as acute lymphoblastic leukemia (ALL) and acute myeloid leukemia (AML) (17, 94–96). These latter studies use bone marrow stromal cells and mesenchymal stem cells (MSCs) as mitochondria donor cells, which develop TNTs to vehicle mitochondria into hematological tumors. Following mito-transfer, recipient leukemic cells exhibit similar features to our experiments with PMPs, in terms metabolic reprogramming, enhanced ROS production, and chemoresistance (17, 94–96). Another study from Levoux et al. (2021) has examined the effects of platelet-derived mitochondria on MSCs growth and wound healing (97). Accordingly, they show that the therapeutic efficiency of MSCs in tissue injury mice models is enhanced following the treatment of platelet-derived mitochondria (97). Despite these interesting and promising results, more research is needed to further elucidate the repercussions and outcome of PMP-mediated (notably mitochondria function) effects in cancer malignancy and disease progression.

One area of study which has recently received growing attention is the role of OXPHOS activation in cancer cell survival and chemoresistance (98). Interestingly, it seems that cancer development mostly relies on the OXPHOS pathway, rather than ATP generation, by facilitating anabolism through anaplerosis to generate ROS intermediates (99). Accordingly, our data show that PMP-mediated OXPHOS enhancement in CLL cells improves leukemia resistance to commonly used drugs Cytarabine, Venetoclax, and Plumbagin. One study analyzing primary pancreatic acinar formation in mice has shown that metabolic-reprogramming and OXPHOS activation in these animals could be the result of dysregulated oncogenic KRAS (100). Moreover, it has been demonstrated that OXPHOS suppression in AML-engrafted mice can elevate cancer cell sensitivity to anti-BCL2 drug Venetoclax and Cytarabine (101). Furthermore, they also found induced levels of mitochondrial mass and functions in CLL cells in response to the BCL-2 inhibitor drug Venetoclax. Mechanistically, they found that mitochondrial reprogramming essential for chemoresistance was mediated by MCL-1 upregulation, which led to AMP-activated protein kinase (AMPK) signaling and subsequent OXPHOS activation (102). Altogether, these studies suggest that increased mitochondrial OXPHOS activity has a crucial role in CLL survival and drug resistance. Therefore, combining the current chemotherapeutic treatment regimens with compounds targeting mitochondrial functions may provide an effective therapeutic strategy for leukemia.

Through a bioinformatics approach, we reached out to a set of metabolic-related genes in PMP-recipient CLL cells which were very similar to what was seen in cancer cells collected from CLL patients (**Figure 4** and **Supplementary Figure 3**). Further analysis showed that this network could cause metabolic reprogramming that improved our cell model’s drug resistance and survival by stimulating the oncogenic factor HIF-1. HIF-1 is a transcription factor that plays a central role in cellular adaptation to low oxygen levels (hypoxia) and regulates the expression of various genes involved in angiogenesis, metabolism reprogramming, and cell survival (103, 104). It has been well presented before that an increasing number of mitochondrial populations can potentially stimulate HIF-1 expression in cancer cells through a complex interplay of cellular signaling pathways (105). It is also vital in coordinating the interaction between neoplastic B cells and their microenvironments (106). Interestingly, the expression of HIF-1 can be upregulated by mitochondrial overgrowth and function in cells through ROS production (107). Our data showed significant ROS generation in CLL cells under PMP stimulation (**Figures 3C, D**). Since mitochondria are a major source of ROS generation in cells, increased mitochondrial mass or activity can result in higher levels of ROS, which in turn can stabilize HIF-1α, one of the subunits of HIF-1, by inhibiting its degradation and lead to increased HIF-1 activity and gene expression (108). The other positive impact of mitochondria enhancement on HIF-1 expression is through ETC activity which can affect cellular oxygen consumption (as already depicted in **Figure 2**) and result in the accumulation of hypoxia-inducible factors (**Figure 5**). Accordingly, a higher mitochondrial population or enhanced ETC activity may lead to an imbalance between oxygen supply and demand, triggering HIF-1 expression (109). The third possible mechanism could be through increasing the cellular metabolites like glucose and glutamine metabolism (110, 111). Changes in mitochondrial function, such as an increase in mitochondrial population, may affect the metabolic profile of cancer cells and alter the metabolism (105), which in turn, can result in the accumulation of metabolites such as succinate or fumarate (produced by SDH enzyme), which have been shown to stabilize HIF-1α and promote HIF-1 expression (105, 112). The last known possible mechanism can be the mitochondrial dynamic changes in morphology, fusion, and fission processes (113). These dynamics are regulated by specific proteins like MCL-1 (114) and result in increased HIF-1 expression (113). Despite these findings, it is important to note that the relationship between mitochondrial population and HIF-1 expression in cancer cells is complex and context-dependent. Further research is needed to elucidate the precise mechanisms underlying this relationship and identify potential therapeutic strategies targeting mitochondrial-HIF-1 interactions in CLL.

Altogether, our results strongly suggest a role for PMPs in CLL development and progression. These findings are also in line with others stating the possibility of PMP involvement in the development of primary tumors and malignancy processes (115, 116). We believe that the distinctive role of platelets in the metabolic remodeling of cancer cells (especially in advanced stages) has been underestimated, notably through PMP-mediated trafficking. This study was the first to demonstrate the impactful role of PMPs as mitochondria delivering vehicles to modulate leukemia pathogenesis. Further characterization of PMP mito-transfer may provide new avenues for strategic intervention. For example, blockade of PMP interactions may amputate cancer cell access to valuable mitochondrial content and functions essential for disease progression. More importantly, our results provide further evidence for the use of antiplatelet treatments in combination with conventional leukemia therapy to attenuate cancer progression. Further research in this area could pave the way for the design of new treatment strategies based on the targeting of cancer metabolism, particularly on mitochondrial-dependent mechanisms.

## Data availability statement

The original contributions presented in the study are included in the article/**Supplementary Material**. Further inquiries can be directed to the corresponding author.

## Ethics statement

The studies involving human participants were reviewed and approved by Institutional Review Board Statement and approval for studies involving human samples have been granted from both the Vitalité Health Network (project: CER7-3-17 Ver. 5) and the Université de Moncton (NB, Canada) (project: 1516-002) research ethics boards. The patients/participants provided their written informed consent to participate in this study.

## Author contributions

Conceptualization, GR, LB, and NP. Study design and Methodology, EG and VV. Bioinformatics, and Statistical analysis, EG. Data Curation and Validation, EG. Writing of original draft, EG and GR. Review and Editing, EG, GR, VV, LB, and NP. Visualization, EG. Supervision, GR, LB, and NP. Funding acquisition, GR, LB, and NP All authors contributed to the article and approved the submitted version.

## Supplementary material

Supplementary Figure 1 Interaction analysis of CLL cells with purified Set2-derived mitochondria. **(A)** Flow cytometry was performed in CLL cell lines labeled with anti-human CD19 antibody either untreated (control) or co-incubated with purified mitochondria derived from the Set2 megakaryocytic cell line labeled with MitoTracker Deep Red (MTDR). Co-incubations of CII (upper panels) or MEC-1 cells (lower panels) with purified mitochondria were performed at 1:10 and 1:100 (cell/mito) ratios for 48 hours. **(B)** CLL co-incubations (48 hours) with purified mitochondria (1:10 and 1:100) were also photographed by fluorescence microscopy. CLL cell lines were labeled with DAPI and MitoTracker™ Green FM (MTG), whereas purified mitochondria were labeled with MTDR. **(C)** Relative mitochondrial DNA copy number was obtained from cell models co-cultured with Set2-Mitos (for 24 and 48 hours) using Real-time PCR on leucine tRNA (mtDNA-tRNA-Leu). Results were normalized by nuclear DNA to reveal relative mitochondrial DNA copy number. Each value is the mean ± SEM of six separate experiments. The asterisk (*) indicates significantly different (**p* < 0.05, ***p* < 0.01, ****p* < 0.001).

Supplementary Figure 2 Impact of damaged mitochondria acquisition on CLL viability. CLL cell lines were evaluated for cell viability at varying co-culture ratios (1:10 and 1:100) and incubation time (24 and 48 hours) with either **(A)** frozen-thawed PMPs or **(B)** PMPs treated with Rotenone (ROT) and Antimycin A (AmA). Freeze-thaw cycling began at -6.5°C and the PMPs were cooled down to -80°C and kept 24 hours, and then warmed up to 37°C in water bath (Repeated 3 times). ROT/AmA treated PMPs were made by incubating the PMPs with 0.5µM ROT (to inhibit complex I) and 2.5µM AmA to block complex III for 1 hour. Viability was then assessed in relation to control non-treated cells (24 h). Each value is the mean ± SEM of six separate experiments. The asterisk (*) indicates significantly different (**p* < 0.05, ****p* < 0.001).

Supplementary Figure 3 Bioinformatics identification and analysis of core metabolic-related genes involved in CLL progression. **(A)** Identification of genes involved in CLL metabolism. The interaction between genes was visualized by the Cytoscape plugins BisoGenet and the hubs were plotted by the CentiScaPe based on their betweenness centrality. **(B)** Enrichment analysis of metabolic related signaling pathways were then configured based on identified genes (Hubs) using EnrichR. The asterisk (*) indicates significantly different (* *p* < 0.05, ***p* < 0.01, ****p* < 0.001).

Supplementary File 4 Metabolic gene expression values in CLL clinical samples. The expression levels of genes associated to metabolic pathways were applied to a cohort of CLL patients (n=442) based on their corresponding Binet staging (i.e., stage A (n)=184, stage B (n)=179, and stage C (n)=79) to define the most significant modulated genes associated with CLL disease progression. The average expression values of the profiled genes are listed per patient.

Supplementary Figure 5 Impact of Set2-derived mitochondria on metabolic gene expression in CLL cells. Real-time PCR analysis was performed to evaluate differentially expressed metabolic-related genes (DEGs) in **(A)** CII and **(B)** MEC-1 cells in the presence of chemo-drugs where each column represents the Set2-Mito treated/non-treated cell ratio. Control groups were treated with DMSO as dilution buffer. Results are expressed as the mean ± SEM of six biological experiments. One-way ANOVA followed by Tukey’s multiple comparisons test show significant differences in the values presenting different superscript letters (*p* < 0.05).

Supplementary Figure 6 Effects of Set2-derived mitochondria on CLL survival and apoptosis. CLL cells were co-incubated with Set2-Mitos (1:100, 48 h) prior to treatments with the cells’ respective LD50 of Cytarabine, Venetoclax, and Plumbagin for 24 hours for the evaluation of drug cytotoxic events. Flow cytometry analysis in **(A)** CII and **(B)** MEC-1 cells stained with Propidium Iodide (PI, X‐axis) and Annexin‐FITC (X‐axis) sorted cell populations according to distinctive quarter zones (Q), including the large nuclear fragments/debris or Q1 (Anx^-^/PI^+^), necrosis cells or Q2 (Anx^+^/PI^+^), apoptotic cells or Q3 (Anx^+^/PI^-^), and the living cells or Q4 (Anx^-^/PI^-^). Values for each quadrant were then plotted in relation to their respective control groups (DMSO). Each value is the mean ± SEM of six separate experiments. The asterisk (*) indicates significantly different (**p* < 0.05, ****p* < 0.001).

Supplementary Figure 7 Impact of Set2-derived mitochondria on CLL cycle progression. CLL cells were co-incubated with Set2-Mitos (1:100, 48 h) prior to treatments with the cells’ respective LD50 of Cytarabine, Venetoclax, and Plumbagin for 24 hours then evaluated for cycle analysis using flow cytometry. Cell accumulation in each cell cycle phase (i.e., <G0/G1, G0/G1, S, G2/M, and >G2/M) for **(A)** CII and **(B)** MEC-1 cells stained with Propidium Iodide and RNase A was then plotted in relation to control groups treated with DMSO. Each value is the mean ± SEM of six separate experiments. The asterisk (*) indicates significantly different (**p* < 0.05, ***p* < 0.01, ****p* < 0.001).

Supplementary Figure 8 Effect of Set2-derived mitochondria on CLL malignant processes. CLL cells were co-incubated with Set2-Mitos (1:100, 48 h) prior to treatments with the cells’ respective LD50 of Cytarabine, Venetoclax, and Plumbagin for 24 hours then evaluated for **(A)** migration and **(B)** invasion capacity using 24-well ThinCerts. Control groups were treated with DMSO as dilution buffer. Each value is the mean ± SEM of six separate experiments. One-way ANOVA followed by Tukey’s multiple comparisons test show significant differences in the values presenting different superscript letters (*p* < 0.05).
