# Supplementary figures and images for: Platelet-derived microparticles provoke chronic lymphocytic leukemia malignancy through metabolic reprogramming
Source: Front Immunol. 2023 Jun 27;14:1207631. doi: 10.3389/fimmu.2023.1207631 (PMC10333545; doi:10.3389/fimmu.2023.1207631)

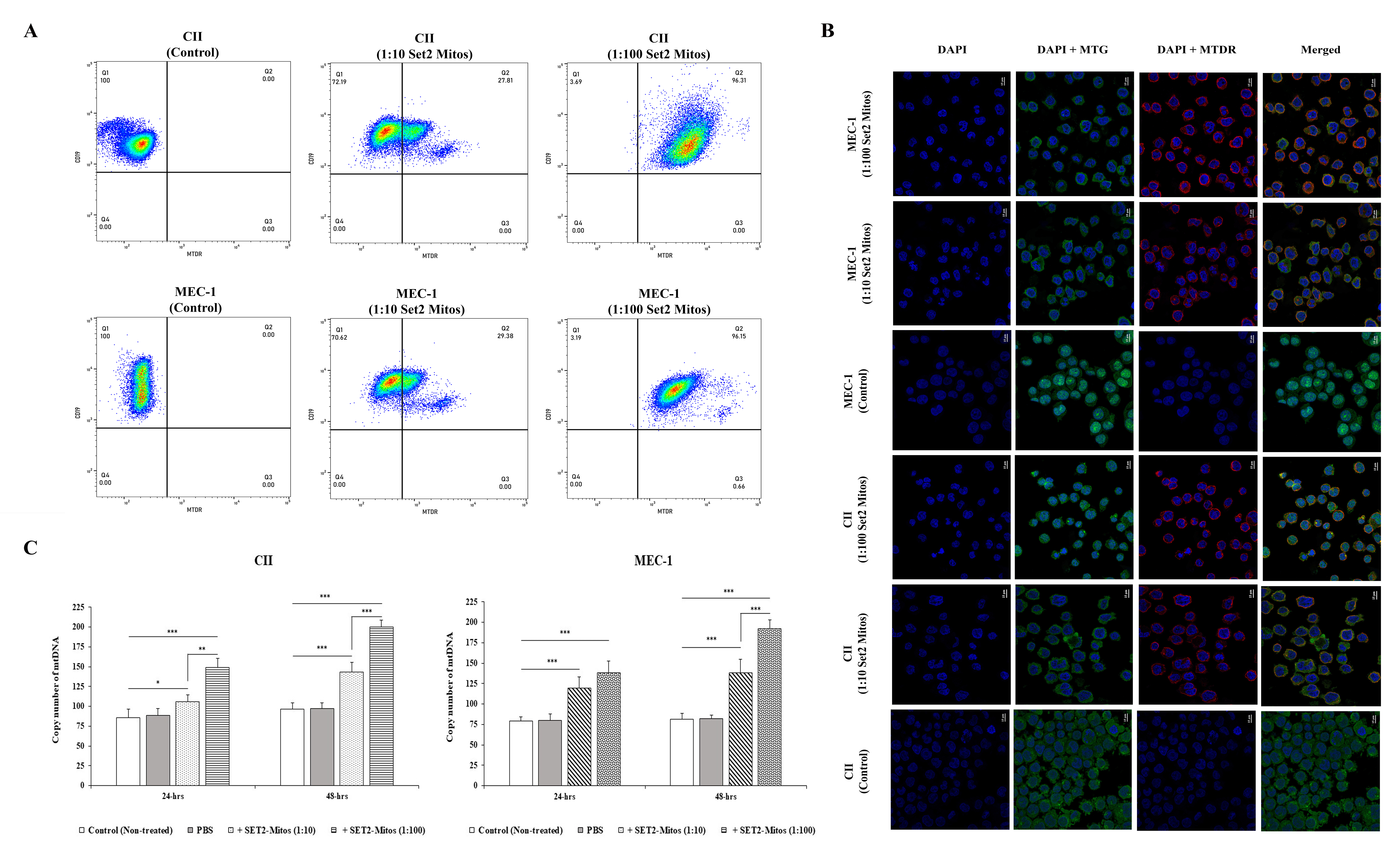

Supplement: Supplementary Figure 1 — Interaction analysis of CLL cells with purified Set2-derived mitochondria. (A) Flow cytometry was performed in CLL cell lines labeled with anti-human CD19 antibody either untreated (control) or co-incubated with purified mitochondria derived from the Set2 megakaryocytic cell line labeled with MitoTracker Deep Red (MTDR). Co-incubations of CII (upper panels) or MEC-1 cells (lower panels) with purified mitochondria were performed at 1:10 and 1:100 (cell/mito) ratios for 48 hours. (B) CLL co-incubations (48 hours) with purified mitochondria (1:10 and 1:100) were also photographed by fluorescence microscopy. CLL cell lines were labeled with DAPI and MitoTracker™ Green FM (MTG), whereas purified mitochondria were labeled with MTDR. (C) Relative mitochondrial DNA copy number was obtained from cell models co-cultured with Set2-Mitos (for 24 and 48 hours) using Real-time PCR on leucine tRNA (mtDNA-tRNA-Leu). Results were normalized by nuclear DNA to reveal relative mitochondrial DNA copy number. Each value is the mean ± SEM of six separate experiments. The asterisk (*) indicates significantly different (*p < 0.05, **p < 0.01, ***p < 0.001). [file Image_1.jpg]

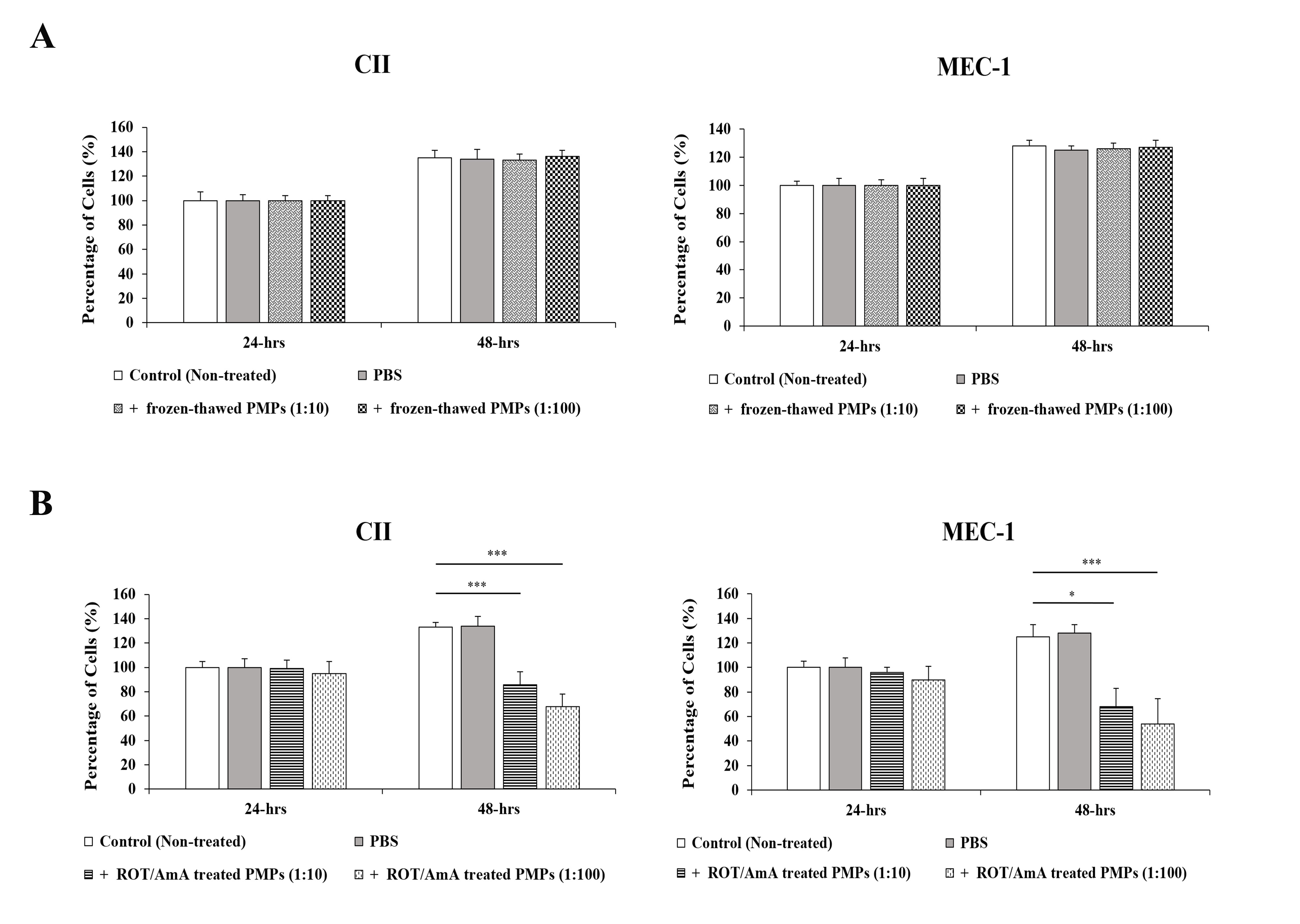

Supplement: Supplementary Figure 2 — Impact of damaged mitochondria acquisition on CLL viability. CLL cell lines were evaluated for cell viability at varying co-culture ratios (1:10 and 1:100) and incubation time (24 and 48 hours) with either (A) frozen-thawed PMPs or (B) PMPs treated with Rotenone (ROT) and Antimycin A (AmA). Freeze-thaw cycling began at -6.5°C and the PMPs were cooled down to -80°C and kept 24 hours, and then warmed up to 37°C in water bath (Repeated 3 times). ROT/AmA treated PMPs were made by incubating the PMPs with 0.5µM ROT (to inhibit complex I) and 2.5µM AmA to block complex III for 1 hour. Viability was then assessed in relation to control non-treated cells (24 h). Each value is the mean ± SEM of six separate experiments. The asterisk (*) indicates significantly different (*p < 0.05, ***p < 0.001). [file Image_2.jpeg]

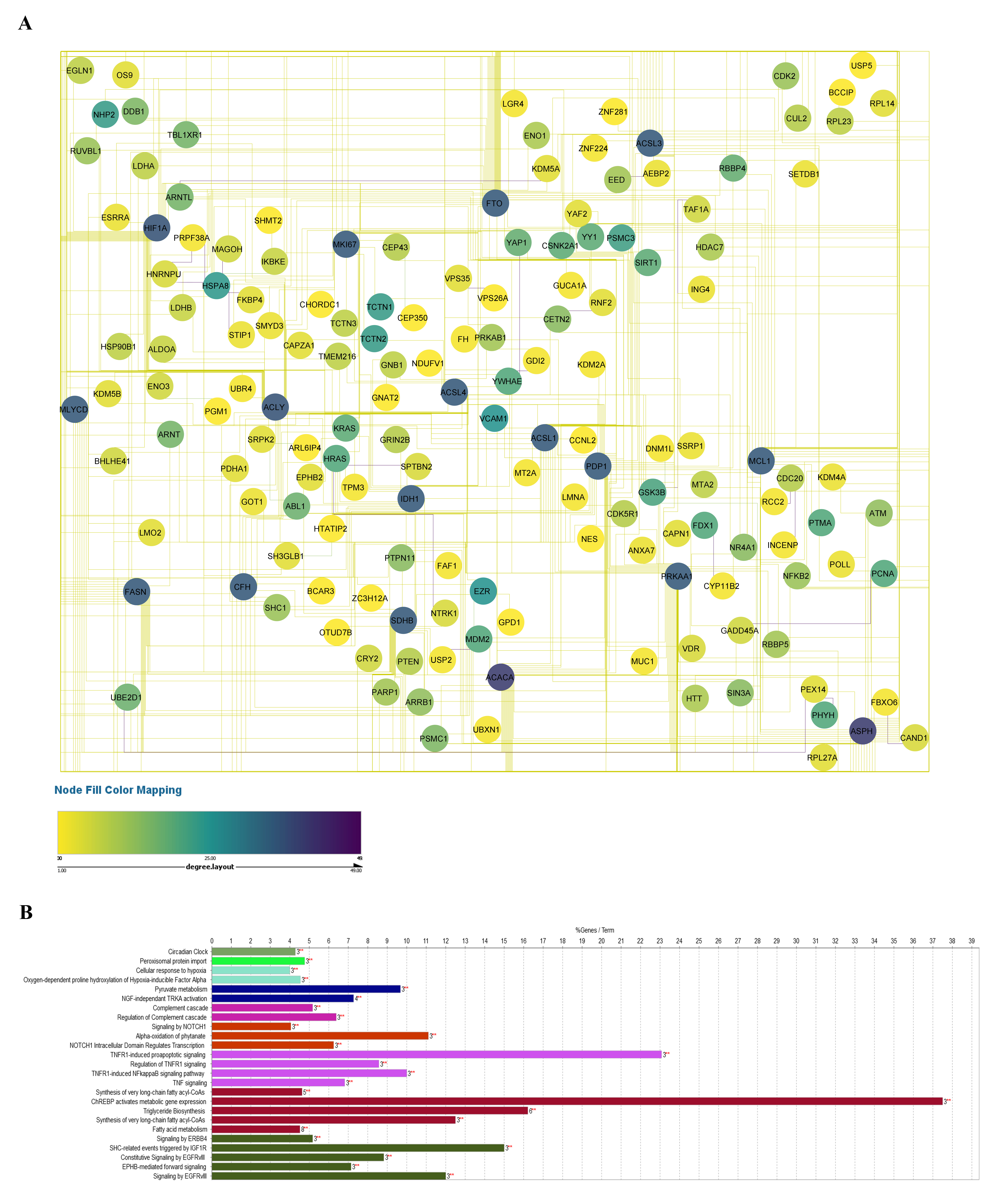

Supplement: Supplementary Figure 3 — Bioinformatics identification and analysis of core metabolic-related genes involved in CLL progression. (A) Identification of genes involved in CLL metabolism. The interaction between genes was visualized by the Cytoscape plugins BisoGenet and the hubs were plotted by the CentiScaPe based on their betweenness centrality. (B) Enrichment analysis of metabolic related signaling pathways were then configured based on identified genes (Hubs) using EnrichR. The asterisk (*) indicates significantly different (* p < 0.05, **p < 0.01, ***p < 0.001). [file Image_3.jpeg]

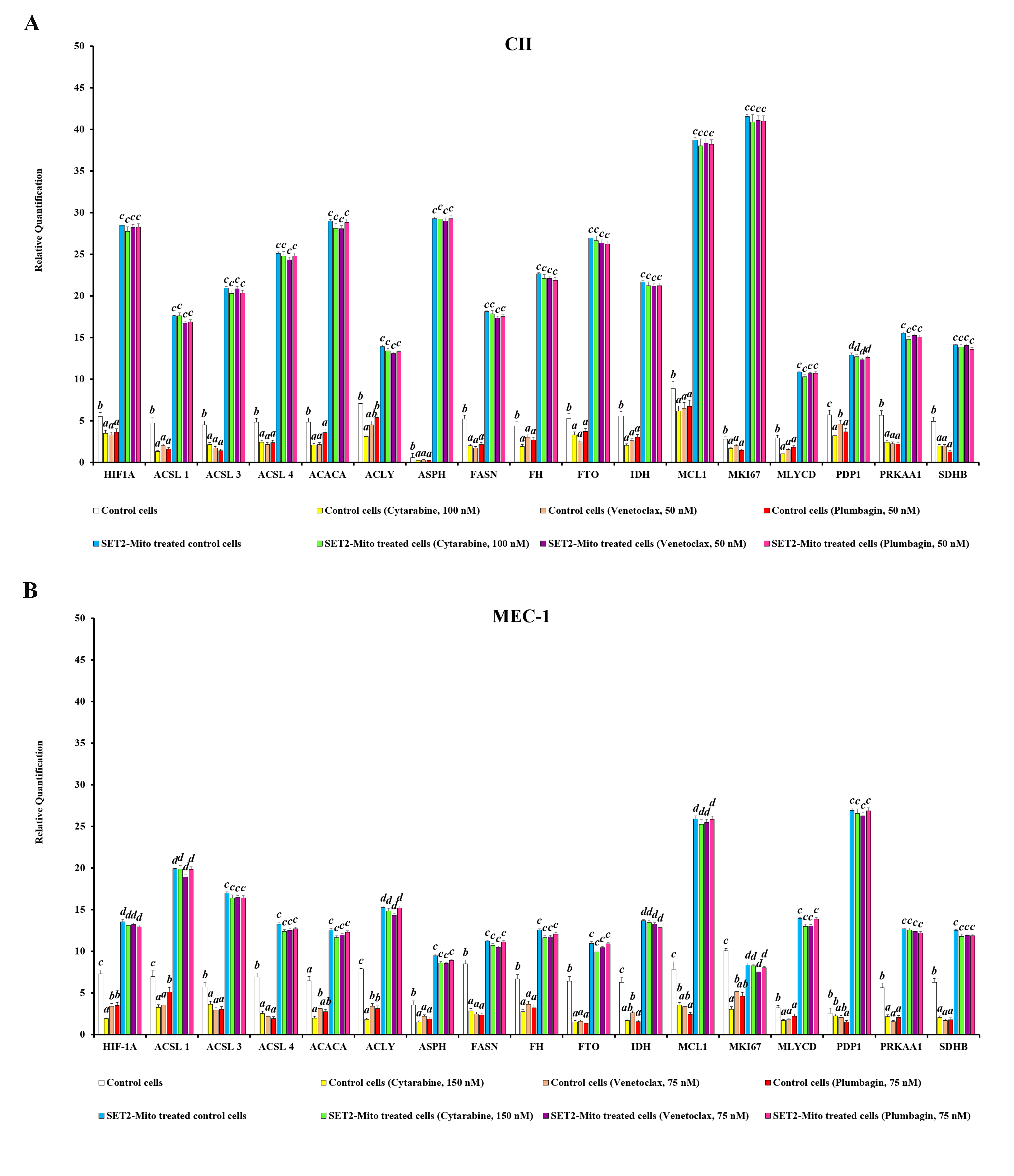

Supplement: Supplementary File 4 — Metabolic gene expression values in CLL clinical samples. The expression levels of genes associated to metabolic pathways were applied to a cohort of CLL patients (n=442) based on their corresponding Binet staging (i.e., stage A (n)=184, stage B (n)=179, and stage C (n)=79) to define the most significant modulated genes associated with CLL disease progression. The average expression values of the profiled genes are listed per patient. [file Image_4.jpeg]

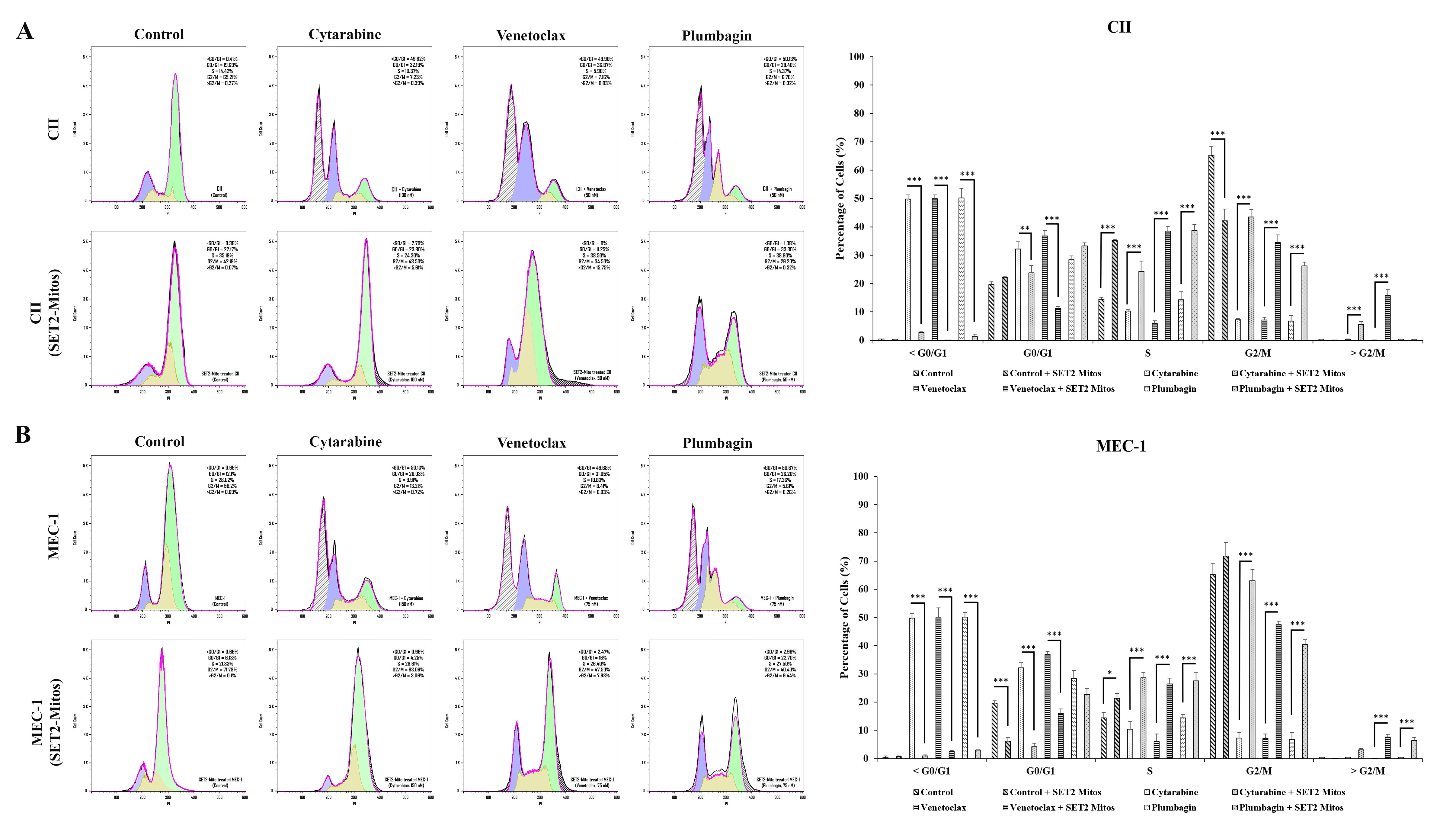

Supplement: Supplementary Figure 6 — Effects of Set2-derived mitochondria on CLL survival and apoptosis. CLL cells were co-incubated with Set2-Mitos (1:100, 48 h) prior to treatments with the cells’ respective LD50 of Cytarabine, Venetoclax, and Plumbagin for 24 hours for the evaluation of drug cytotoxic events. Flow cytometry analysis in (A) CII and (B) MEC-1 cells stained with Propidium Iodide (PI, X‐axis) and Annexin‐FITC (X‐axis) sorted cell populations according to distinctive quarter zones (Q), including the large nuclear fragments/debris or Q1 (Anx-/PI+), necrosis cells or Q2 (Anx+/PI+), apoptotic cells or Q3 (Anx+/PI-), and the living cells or Q4 (Anx-/PI-). Values for each quadrant were then plotted in relation to their respective control groups (DMSO). Each value is the mean ± SEM of six separate experiments. The asterisk (*) indicates significantly different (*p < 0.05, ***p < 0.001). [file Image_6.jpeg]

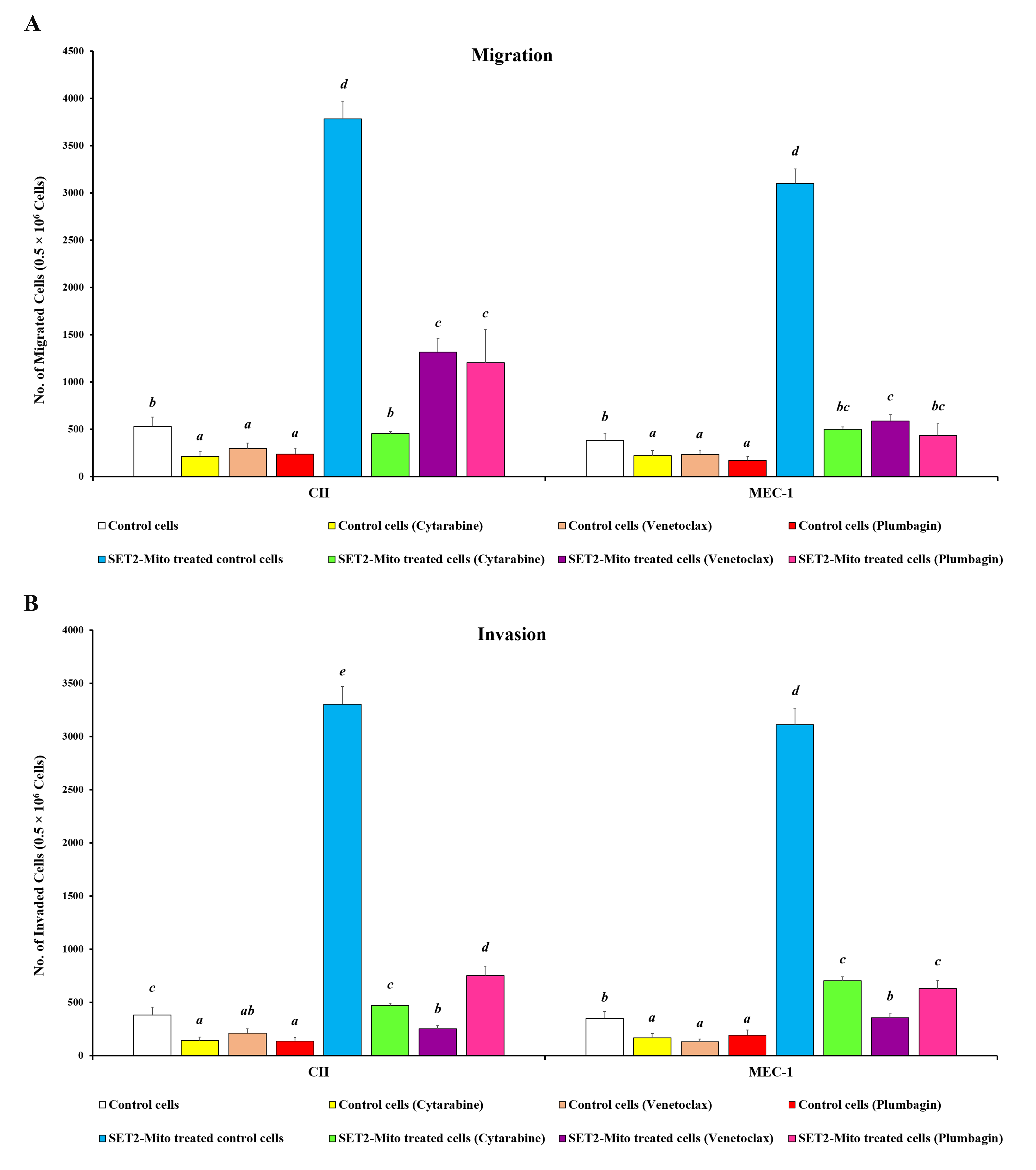

Supplement: Supplementary Figure 7 — Impact of Set2-derived mitochondria on CLL cycle progression. CLL cells were co-incubated with Set2-Mitos (1:100, 48 h) prior to treatments with the cells’ respective LD50 of Cytarabine, Venetoclax, and Plumbagin for 24 hours then evaluated for cycle analysis using flow cytometry. Cell accumulation in each cell cycle phase (i.e., <G0/G1, G0/G1, S, G2/M, and >G2/M) for (A) CII and (B) MEC-1 cells stained with Propidium Iodide and RNase A was then plotted in relation to control groups treated with DMSO. Each value is the mean ± SEM of six separate experiments. The asterisk (*) indicates significantly different (*p < 0.05, **p < 0.01, ***p < 0.001). [file Image_7.jpeg]
